# Chirped Fiber Bragg Grating-Based Cavity Sensor for Simultaneous Temperature and Refractive Index Sensing

**DOI:** 10.3390/s26175647

**Published:** 2026-09-05

**Authors:** Shakhrizat Alisherov, Aidana Bekenkali, Sabina Sairangazy, Zhalgaskhan Bakhytbek, Toheeb Olalekan Oladejo, Sabira Seipetdenova, Daniele Tosi, Carlo Molardi

**Affiliations:** 1 School of Engineering and Digital Sciences, Nazarbayev University, 010000 Astana, Kazakhstandaniele.tosi@nu.edu.kz (D.T.); 2 Laboratory of Biosensors and Bioinstruments, National Laboratory Astana, 010000 Astana, Kazakhstan

**Keywords:** fiber optic sensor, chirped fiber Bragg gratings, dual-parameter sensing, thermo-refractometric sensor, biosensing, biomarker detection

## Abstract

In this work, a compact dual-parameter fiber optic sensor for temperature, refractive index, and label-free biomarker-related measurements is presented. The sensor is based on a chirped fiber Bragg grating cleaved inside the grating region, leaving residual grating lengths of 3 mm, 4 mm, and 5 mm. After cleaving, the reflected spectrum becomes narrower and forms clear sidelobes, allowing the main-band wavelength shift to be used for temperature monitoring, while the sidelobe intensity variation is used for refractive index detection. Temperature calibration from 25 °C to 80 °C showed approximately linear responses with sensitivities close to 10 pm/°C. Refractive index calibration in 10–15% sucrose solutions showed a monotonic decrease in sidelobe intensity with increasing refractive index, with sensitivities reaching about −220 dB/RIU for selected valley features and around −200 dB/RIU in the matrix analysis. An extended calibration from 0% to 35% sucrose confirmed that the RI-dependent response was maintained over a broader range, although with increased nonlinearity. Cross-sensitivity coefficients were incorporated into 2 by 2 sensitivity matrices and their inverses for simultaneous parameter reconstruction. Additional measurements under combined temperature and sucrose concentration changes demonstrated temperature reconstruction with RMSE values of 0.87–2.69 °C, depending on the sensor and experimental conditions. Finally, as a proof-of-concept experiment, the cleaved-CFBG sensors were biofunctionalized with anti-VEGF antibodies and tested for VEGF detection from 10^−15^ M to 10^−10^ M. Overall, the proposed design is simple, compact, compatible with standard interrogation systems, and shows promise as a thermo-refractometric platform for biomedical biosensing applications.

## 1. Introduction

Fiber optic sensors (FOSs) have been significantly developed over the last few decades, making them more useful in a wide variety of applications. FOSs, built to measure multiple physical parameters, have become an important aspect of optical fiber technology. Unlike conventional sensing techniques, FOSs are the most appropriate choice for applications where traditional sensors are ineffective. FOSs offer a large number of advantages which directly derive from their unique physical and operational characteristics [1,2].

FOSs have the advantage of being compact and lightweight, which makes them suitable for scenarios with constraints on space and weight [3,4,5,6,7]. Their flexibility in design enhances their versatility for different application scenarios and configurations [8,9,10,11]. This is supported by low optical losses and high bandwidth capabilities, which allow for efficient signal transmission and wide frequency operation. Another important benefit is their immunity to Electromagnetic Interference (EMI) which allows their use in Magnetic Resonance (MR) applications [12,13,14,15].

FOSs are also highly valued for their high sensitivity and fast response, which make them suitable for in situ sensing and real-time measurements [8,9,12,15]. They are able to work in harsh environments, and they present versatility for many applications in terms of durability, lifespans and resistance to corrosion. They are non-toxic, chemically inert and biocompatible, which makes them suitable for medical applications [3,6,13].

Given these multiple advantages, FOSs are widely utilized for measuring physical parameters, such as temperature and strain, pressure and vibration. In addition, FOSs can be adapted for refractive index (RI) sensing through suitable modification of the fiber structure, such as tapering, etching, or other approaches that enhance the interaction between the guided light and the surrounding medium [3,6,10,16]. With appropriate surface functionalization, RI-based FOSs can further be converted into biosensors, enabling the selective detection of proteins, DNA, infections, and biomarkers, as well as applications in drug-delivery monitoring, water-quality assessment, and pollution detection. These advantages make FOSs suitable for a wide range of sensing applications in industrial manufacturing, aerospace, environmental monitoring, chemical engineering, healthcare and medicine [1,7,15,17]. Moreover, FOS-based biosensors can also be integrated with lab-on-chip systems, where sample handling, processing and analysis are combined in a single compact platform [18,19].

The main technology that enables massive use of FOS is fiber Bragg grating (FBG), which consists of a periodic modulation of the core RI that reflects a narrow spectral band centered at the Bragg wavelength. Changes in strain, temperature, pressure or other physical parameters alter this wavelength, thus making it possible to accurately sense the changes through wavelength demodulation of the reflected spectrum [2,10,20]. Multiple FBGs with different center wavelengths can be inscribed in a single optical fiber. This allows wavelength division multiplexing (WDM) where multiple sensing points can be monitored simultaneously, by forming a sensing network [8,9,21,22]. Compared with alternative FOS techniques, such as surface plasmon resonance and Fabry–Perot interferometry, FBG-based sensors offer several advantages, including compact structure, wavelength-encoded response, high repeatability, and convenient multiplexing capability. However, standard FBGs are mainly sensitive to temperature and strain, while RI sensing usually requires modification of the fiber or grating structure [6,17,23].

The performance of FBGs can be improved by modifying the grating pattern to produce non-uniform gratings. By changing the grating type, profile, or chirp, it is possible to modify the spectral response. Other improvements are obtained by using different fabrication methods such as etching and tilting [24,25,26]. FBGs and arrays of FBGs, given their peculiar properties of biocompatibility, small size, and great spatial resolution are particularly interesting within the biomedical industry and show potential as biosensors [12,13].

Chirped FBGs (CFBGs) have a varying pitch along the length of the fiber. This results in a variation in the Bragg wavelength along the grating, such that different segments reflect different wavelengths [12,21,27]. As a result, CFBGs behave like a chain of FBGs and produce a broad reflection spectrum. This characteristic makes CFBGs act as semi-distributed sensors. Therefore, strain and temperature variations can be detected along their length [7,8,9,21]. Similar to conventional FBGs, CFBGs enable spatiotemporal monitoring of strain and temperature via standard interrogation systems, using their reflection spectra for spatially resolved measurements. Because of these capabilities, CFBGs are also used in various fields, including mechanical engineering and healthcare [6,9,27].

In recent years, procedures like thermal ablation have gained prominence for treating tumors [13]. Accurate temperature feedback defines end points for complete ablation, reduces recurrence, and strengthens hyperthermia treatment planning [8]. While temperature monitoring is central to therapy control, additional diagnostic value can be achieved through RI sensing. RI sensors operate either as bare refractometers, or as functionalized devices for selective biomolecule or cell detection in immunosensors, enabled by thin metallic films or silanization [3]. Hence, a sensor that can sense both RI and temperature, i.e., a thermo-refractometer, is needed [12]. Simultaneous temperature and RI sensing can be useful in many applications including radiofrequency ablation, laser ablation, real-time blood temperature monitoring in angioplasty, and epidural anesthesia [6].

Several strategies have been reported for simultaneous temperature and RI sensing. Within CFBG-based designs, RI sensitivity has been introduced by partial etching [6] or shallow tapering [3]. Other approaches use an etched CFBG Fabry–Perot interferometer in multicore fiber [28], a supermode interferometer [24], or cascaded tilted-grating and FBG/CFBG configurations [15,25]. These methods differ in the physical modification used to create RI sensitivity and in the spectral observable used to distinguish RI from temperature. Table 1 compares representative approaches with the cleaved-CFBG sensor presented in this work.

The comparison shows that the RI-sensitive channel in previous dual-parameter sensors has been created through chemical etching, fiber tapering, multicore-fiber interferometry, or the addition of tilted and Bragg grating elements. In the present approach, RI sensitivity is obtained by cleaving a single CFBG within the grating region. Cleaving narrows the main reflection band and produces pronounced sidelobes, providing two distinct sensing observables: temperature is measured from the wavelength shift, whereas RI is measured from the sidelobe intensity change. Thus, the proposed approach enables simultaneous temperature and RI sensing using a compact single-CFBG architecture without chemical etching, tapering, multicore fiber, or additional grating elements.

Accordingly, this work investigates three cleaved-CFBG sensors with residual grating lengths of 3, 4, and 5 mm. Their temperature and RI responses are calibrated independently, and cross-sensitivity between the two sensing channels is evaluated using a 2 × 2 sensitivity matrix. Monitoring temperature and RI is important in biomedical sensing and biomarker analysis, including applications related to cancer, diabetes, and hemoglobin concentration for anemia diagnosis. In addition, biofunctionalization can be employed to increase sensor specificity toward selected target proteins [29,30,31,32].

In this context, this work introduces a dual-parameter sensing structure based on CFBGs. The proposed sensor is designed to measure both temperature and RI concurrently. Experimental calibration and cross-sensitivity analysis are performed, followed by an inverse sensitivity matrix approach and measurements under simultaneous temperature and RI variations. Finally, anti-VEGF functionalization is used to explore the feasibility of the same sensing platform for preliminary VEGF detection as a proof-of-concept demonstration.

## 2. Materials and Methods

### 2.1. Materials, Setup, and Processing

This study describes a methodology for fabricating a dual-parameter sensor based on CFBGs, using wavelength shift for temperature measurements and an intensity-based metric for RI detection. The optical fiber was cleaved inside the grating region. This left a short section with an exposed tip, which acts as a weak reflector and forms a compact semi-distributed resonator. Temperature variations affect the grating period, shifting the reflected wavelength peak. In contrast, changes in surrounding RI predominantly modulate the reflected intensity at the cleaved sensing tip. Thus, temperature is inferred from the wavelength shift, while RI is quantified using an intensity-based parameter.

Sensor performance was characterized in two stages to isolate temperature and RI responses. For temperature calibration, the CFBGs were immersed in a water-filled flask, and the temperature was increased in controlled steps, while spectral data were recorded at each step. For RI calibration, the sensor was immersed at constant room temperature in aqueous sucrose solutions of varying concentrations. These experiments minimized temperature-induced effects in order to concentrate only on RI sensitivity.

**Figure 1:**
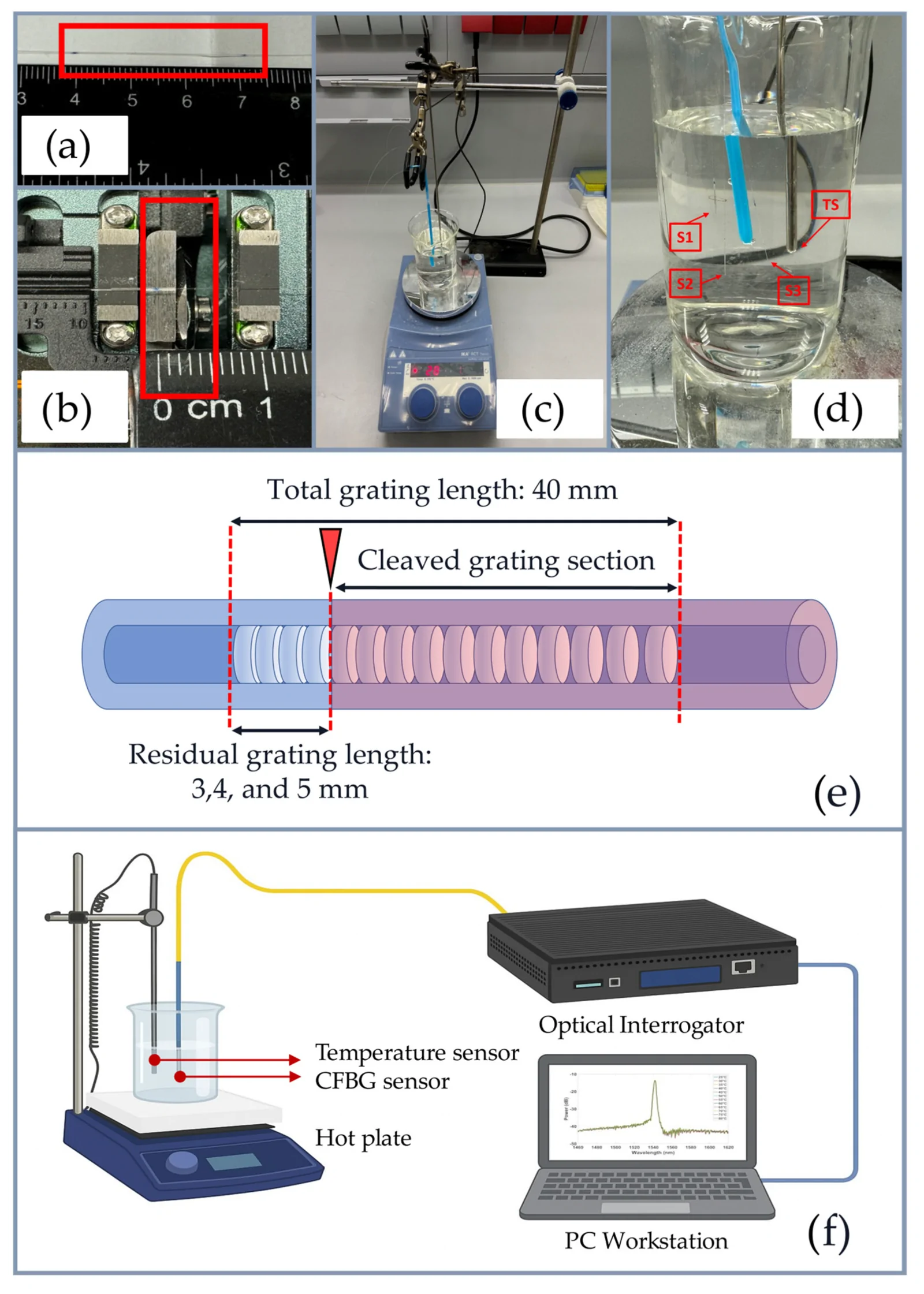
Picture of the sensor preparation and calibration setup: (**a**) CFBG with the 40 mm sensing region highlighted by the red box; (**b**) CFBG positioned for cleaving inside the grating region, leaving a short residual segment highlighted by the red box; (**c**) water-heating setup with the sensors held vertically in the flask; and (**d**) close-up view showing the three cleaved-CFBG sensors (Sensor 1 (S1), Sensor 2 (S2), and Sensor 3 (S3)) and the reference temperature sensor (TS); (**e**) schematic representation of the sensor fabrication concept, showing cleaving inside the grating region and the remaining grating segment, (**f**) schematic representation of the experimental setup used for spectral interrogation and calibration of the cleaved-CFBG sensors.

Spectral data were acquired using the Micron Optics HYPERION si255 optical sensing interrogator (Luna Inc., Atlanta, GA, USA), equipped with 8 optical channels and operating over a wavelength range of 1460–1620 nm. The system enables simultaneous interrogation of multiple fiber optic sensors connected across its channels. Data collection and initial analysis were conducted using the ENLIGHT sensing analysis software (version 1.18.8.0) for optical sensor networks. The obtained datasets were exported to MATLAB (version R2025b), where custom scripts were applied for data normalization, feature extraction, calibration curve fitting and figure generation, followed by further processing and visualization. A schematic view of the experimental setup, sensor and photographs is shown in Figure 1.

Calibration curves were obtained by separately analyzing the wavelength shift against temperature in a water heating experiment and the selected intensity metric against the known RI of sucrose solutions. To address cross-sensitivity, a two-equation sensitivity model was applied. This model relates changes in wavelength and intensity to both temperature and RI. Sensitivity coefficients were obtained from the individual calibration experiments. The resulting 2 × 2 system was inverted to estimate temperature and RI from concurrent measurements. Performance was evaluated for linearity and monotonicity of the calibration curves.

### 2.2. Sensor Fabrication

The optical fibers used in this study were custom manufactured by Technica Optical Components LLC (Tianjin, China), and their main parameters are listed in Table 2.

Three types of CFBG-based thermo-refractometric sensors were fabricated by cleaving the fiber within the grating region, leaving the lower-wavelength side as the sensing tip. To identify the sensitive section before cleaving, gentle, localized pressure was applied along the approximate grating length while the spectral response was monitored with a Micron Optics interrogation system. The applied pressure produced measurable wavelength shifts in the reflected spectrum, after which the start and end points of the target section were identified and marked on the fiber coating. The fiber was then cleaved using a Fujikura fiber cleaver. The cleaved tip formed a cavity in direct contact with the surrounding medium, enhancing dual sensitivity.

In the proposed structure, the term cavity does not refer to a conventional Fabry–Perot cavity formed by two discrete reflectors with a single fixed cavity length. Instead, the cleaved fiber tip provides a weak reflection, while the remaining portion of the CFBG provides a distributed wavelength-dependent reflection. The interference between these contributions produces the post-cleavage sidelobe structure and forms a compact semi-distributed resonator. Since different wavelengths are reflected at different longitudinal positions along the chirped grating, a single effective cavity length cannot be assigned to the complete reflected spectrum. Therefore, the directly defined geometrical parameter of the structure is the residual grating length after cleaving.

In this procedure, three sensors with residual grating lengths of 3 mm, 4 mm, and 5 mm were fabricated for comparison purposes. All sensors were connected to the Micron Optics system for simultaneous spectral acquisition. The resulting data were analyzed in MATLAB: traces were normalized, features were extracted, calibration curves were fitted, and their suitability for temperature and RI sensing experiments was assessed.

### 2.3. Temperature Calibration

To investigate the performance of the fabricated CFBG sensors for detecting temperature change, all sensors were submerged into a laboratory flask containing approximately 200 mL of water at room temperature about 25 °C. A thermocouple connected to the hot plate was immersed in the water to monitor the actual water temperature, while the measured temperature was displayed on the hot plate control panel. The experimental setup was composed of a metal support structure that kept the CFBG sensors and the thermocouple in the same position during the whole experiment in order to ensure their co-location. The flask was then placed on a heating plate, and the water temperature was gradually increased from 25 °C to 80 °C. Spectral responses from all sensors were recorded into separate files at each 5 °C step and the resulting datasets were plotted in MATLAB to visualize the evolution of the spectra with temperature.

### 2.4. Refractive Index Calibration

To assess the sensitivity to RI, the sensors were subsequently immersed in aqueous sucrose solutions, prepared in distilled water, spanning 10.0% to 15.0% in 0.5% increments, yielding eleven concentration levels. To begin with, a 40% sucrose stock solution was prepared by dissolving 40.0 g of sucrose in a small volume of distilled water at room temperature, with continuous stirring until fully dissolved. The final volume was reduced to 100 mL by means of distilled water. From this 40% stock solution, another 50 mL of a 10% stock solution was prepared by mixing 12.5 mL of the 40% sucrose solution with 37.5 mL of distilled water. Then to record baseline spectral responses, all fabricated CFBG sensors were again immersed in a laboratory flask filled with an initial 5 mL sample of 10% sucrose solution at room temperature, approximately 25 °C. The sucrose concentration was subsequently increased in 0.5% steps by adding calculated volumes of the 40% stock to the sample. The required volume was obtained from the standard mixing equation, taking into account the progressively increasing total sample volume. After each addition, the solution was mixed and allowed to stabilize before the sensor spectra were recorded.

In addition, the RI response was evaluated over a wider sucrose concentration range from 0% to 35% with 5% increments. For this experiment, 5 mL of distilled water was used as the initial 0% sucrose condition. The sucrose concentration was then increased by 5% increments by adding the calculated volume of the 40% stock sucrose solution. Consequently, the total solution volume progressively increased with each step. The measurements were performed at the same room temperature, and the sensor spectra were recorded after mixing and stabilization at each concentration. This extended calibration was used to evaluate the overall RI response of the sensors, whereas the 10–15% range remained the primary calibration interval for the sensitivity and cross-sensitivity analysis.

### 2.5. Cross-Sensitivity Formulation

The response of the thermo-refractometer to changes in external parameters such as RI (∆n) and temperature (∆T) is represented by variations in the intensity (∆I) of the observed spectrum and wavelength shift (∆λ) of the main band, respectively. This relationship between external parameters and observed measurements can be mathematically expressed in terms of a 2 × 2 cross-sensitivity matrix as follows:

(1) $$\left[\begin{array}{c} ∆I\\ ∆\lambda \end{array}\right]=\left[\begin{array}{cc} \frac{∆I}{∆n} & \frac{∆I}{∆T}\\ \frac{∆\lambda }{∆n} & \frac{∆\lambda }{∆T} \end{array}\right]\left[\begin{array}{c} ∆n\\ ∆T \end{array}\right],$$

where ∆*I*/∆*n*—RI sensitivity, ∆*λ*/∆*T*—temperature sensitivity, ∆*λ*/∆*n*—cross-sensitivity to RI, and ∆*I*/∆*T*—cross-sensitivity to temperature.

The determinant of the cross-sensitivity matrix was used to evaluate the relative contribution of the cross-sensitivity terms. For the matrix defined in Equation (1),

(2) $$\det\left(S\right)=S_{11}S_{22}-S_{12}S_{21},$$

where *S*_11_ = ∆*I*/∆*n* and *S*_22_ = ∆*λ*/∆*T* represent the primary RI and temperature sensitivities, whereas *S*_12_ = ∆*I*/∆*T* and *S*_21_ = ∆*λ*/∆*n* represent the corresponding cross-sensitivities. When the off-diagonal contributions are small, the determinant approaches the product of the main diagonal terms, *det*(*S*) ≈ *S*_11_ *S*_22_. Therefore, comparison between these two quantities provides an estimate of the relative contribution of cross-coupling between the two sensing channels.

### 2.6. Biofunctionalization

The functionalization of the CFBGs was carried out according to our previously established protocol, with a notable modification in the cleaning process [33]. Instead of using the traditional Piranha solution, an ozone cleaner was used to prepare the fiber tip surfaces. The ozone cleaner was selected because a highly corrosive Piranha solution can damage CFBG sensing sites. The fibers were first ozone-cleaned using the UV/ozone cleaner, ProCleaner™ (Model 220, BioForce Nanosciences Holdings, Inc., Ames, IA, USA), for 15 min. This step eliminated organic contaminants and increased the surface energy of the fiber tip by the formation of hydroxyl groups on the surface. The surfaces of the cleaned fiber tips were then treated with a 1% APTMS (3-aminopropyltrimethoxysilane) and methanol solution for 20 min to silanize the surfaces by attaching amino groups. The attachment of the amino groups increased the likelihood of the cross-linker to bind to the tip surface. This was followed by a methanol rinse and oven baking at 110 °C for 1 h, to promote cross-linking of the silane molecules and to stabilize the monolayer. After that, the fibers were removed from the oven and rinsed with deionized water. The fiber tips were then incubated in 25% glutaraldehyde (GA) in phosphate-buffered saline (PBS) for 1 h, in a MultiTherm Shaker (Model H-5000-HE, Benchmark Scientific Inc., Edison, NJ, USA) with constant stirring at 300 rpm and 25 °C. GA activates the tip surface by forming covalent cross-links between the fiber and antibodies. The fiber tips were incubated for 1 h in a 500 μL solution containing PBS and 8 μg/mL of anti-VEGF antibodies (PAA143Hu01, Cloud-clone Corporation, Houston, TX, USA). The sensor tips were rinsed with PBS to remove unbound antibodies. The unreacted groups were then blocked with mPEG-Amine (methoxypolyethylene glycol amine) for 30 min to reduce non-specific binding and improve sensor selectivity. Rinsing with PBS cleaned the remaining blocking solution and the functionalized CFBG sensors’ tips were stored in a tube containing PBS to maintain hydration and stability until biomarker detection.

### 2.7. Limit of Detection Analysis

For VEGF measurements, the spectral response was evaluated from the intensity changes of the detected sidelobe peaks and valleys relative to the PBS blank condition. The responses were grouped into left peaks, right peaks, left valleys, and right valleys, and the mean response was calculated for each VEGF concentration. The Limit of Detection (LoD) was estimated from the low-concentration region of the calibration response following the calibration-curve approach reported for fiber optic biosensors [34,35]. The first four measured VEGF concentrations, from 10^−15^ M to 10^−12^ M, were fitted using a second-order polynomial as a function of the logarithm of concentration:

(3) $$y\;=f({log}_{10}C),$$

where C is VEGF concentration and y is the baseline-corrected spectral intensity response. The detection threshold was defined as follows:

(4) $$y_{LoD}\;=y_{blank}+{3\sigma }_{max},$$

where $y_{blank}$ is the mean response of the PBS blank and $\sigma_{max}$ is the maximum standard deviation of the repeated measurements among the calibration points considered for the LoD estimation. Since all responses were baseline-corrected to PBS, $y_{blank}=0$. The LoD concentration was then obtained from the intersection of the fitted calibration curve with $y_{LoD}$, equivalently:

(5) $$C_{LoD}\;=f^{-1}(y_{blank}+{3\sigma }_{max}),$$

## 3. Results and Discussion

### 3.1. Spectral Characteristics

The spectral characteristics of all CFBG sensors were evaluated using the Micron Optics interrogation system, both before and after cleaving. Spectral data were processed using ENLIGHT software and visualized in MATLAB.

**Figure 2:**
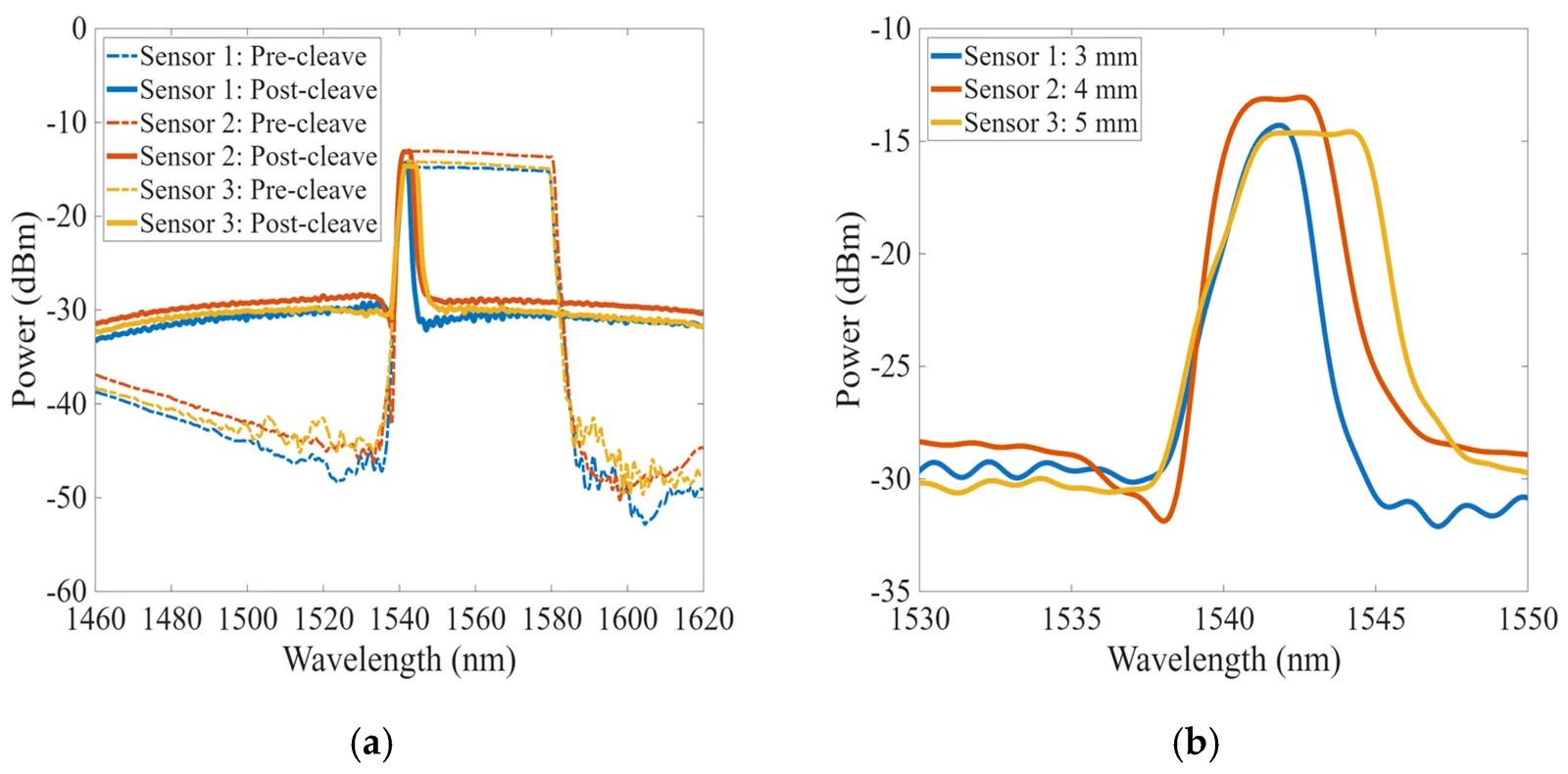
Reflection spectra of the CFBG sensors before and after cleaving: (**a**) full reflected spectra of the three sensors before and after cleaving; (**b**) enlarged view of the post-cleavage main reflection bands corresponding to residual grating lengths of 3 mm, 4 mm, and 5 mm. The post-cleavage spectra show narrowing of the main reflection band and formation of sidelobes used for intensity-based RI interrogation.

Initially, sensors were cleaved near the start of the grating, leaving only 1–2 mm on the lower-wavelength side. However, this approach completely erased the grating, resulting in spectra with no reflection bands. Since light was no longer reflected, the cleaving strategy was modified to retain a longer residual grating segment. Sensors with residual lengths of 3 mm (Sensor 1), 4 mm (Sensor 2), and 5 mm (Sensor 3) were successfully fabricated. The spectra were significantly altered by cleaving, which resulted in a narrower main band surrounded by prominent sidelobes that emerged on both sides, as shown in Figure 2. This transformation enables dual sensing: the narrowed main band is sensitive to wavelength shifts, while sidelobes respond to intensity changes. Thus, a single device can simultaneously measure both parameters.

Figure 2 illustrates that the main reflection bands narrowed after cleaving, and sidelobes became more apparent on both the lower- and higher-wavelength sides. For Sensor 1, cleaved to leave 3 mm of grating on the lower-wavelength side, the broad pre-cleave band (1540–1580 nm) compresses to a narrow band near 1541 nm with sidelobes on both sides after cleaving. Sensor 2 was cleaved, leaving 4 mm of grating on the lower-wavelength side. Its bandwidth shrinks about ten times from roughly 40 nm to roughly 4 nm, and similar low- and high-wavelength sidelobes emerge. Sensor 3 was cleaved, retaining 5 mm of grating. Its original bandwidth is shortened by roughly 85%, producing a spectrum with comparable sidelobes.

After fabrication and calibration, temperature is expected to vary linearly with peak wavelength shift, while sucrose concentration (RI) correlates with sidelobe attenuation. These effects remain decoupled. This behavior is expected for a chirped grating with the formation of a low-reflectivity tip, which, together with the remaining grating, behaves like a semi-distributed resonator. Further experiments showed that the cleaved tip enhances coupling with the surrounding medium, resulting in increased intensity sensitivity to the RI in the sidelobes while retaining wavelength sensitivity to temperature in the main band.

### 3.2. Temperature Sensitivity Analysis

This section presents the results of the temperature experiment. The spectral response of the cleaved CFBGs to temperature variation is very similar to standard FBGs, in which the Bragg wavelength shifts linearly to longer wavelengths with increasing surrounding temperature.

**Figure 3:**
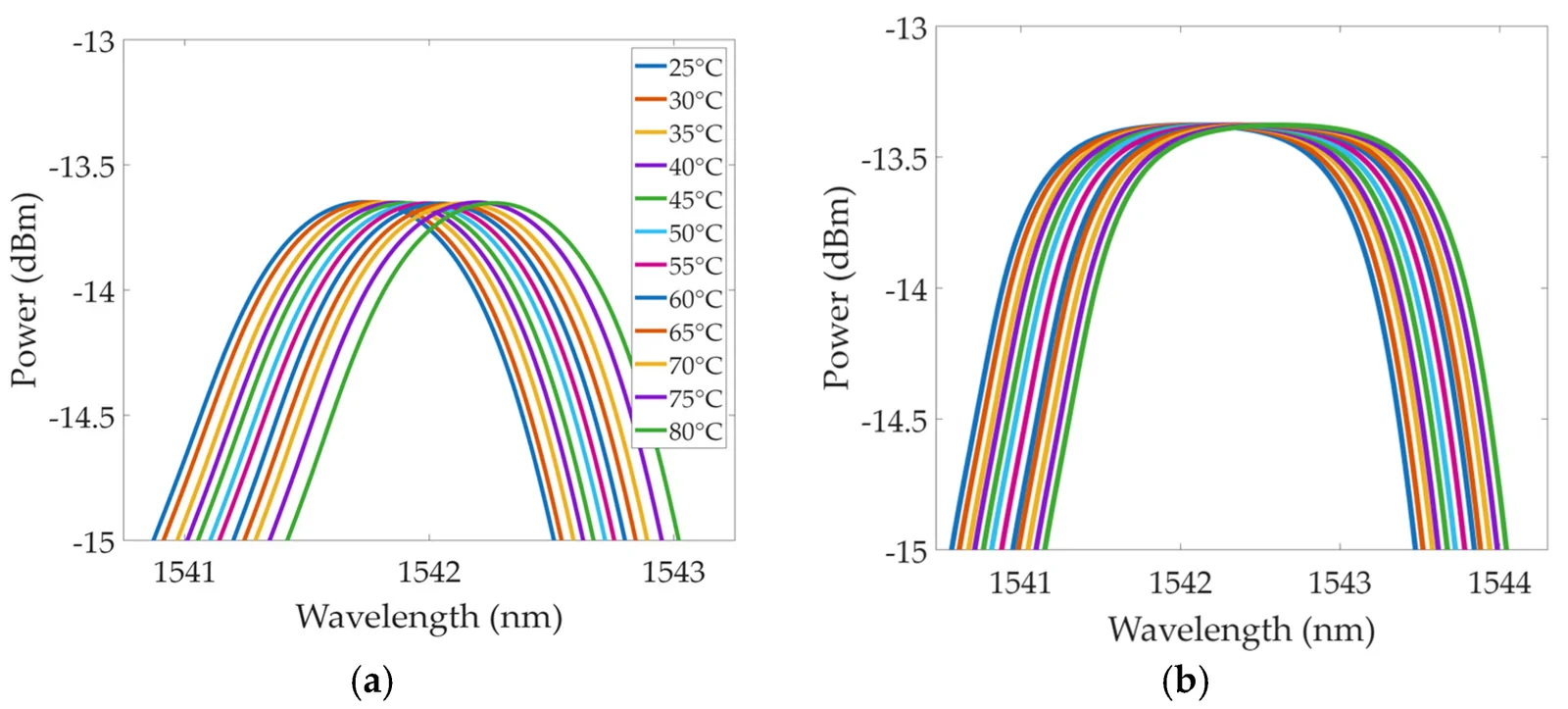

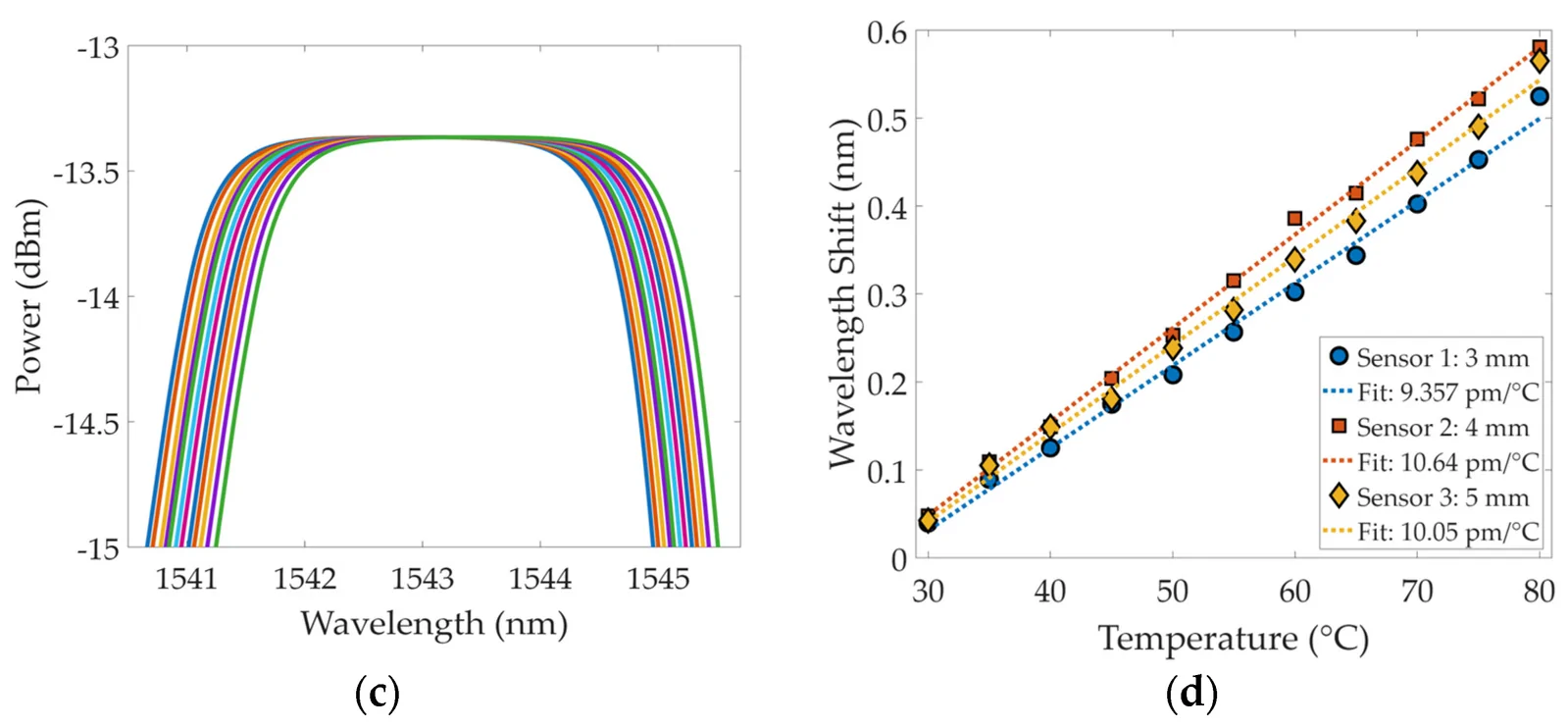
Temperature-dependent reflection spectra of the cleaved-CFBG sensors during heating from 25 °C to 80 °C: (**a**) magnified main reflection band of Sensor 1 with a residual grating length of 3 mm; (**b**) magnified main reflection band of Sensor 2 with a residual grating length of 4 mm; (**c**) magnified main reflection band of Sensor 3 with a residual grating length of 5 mm; (**d**) wavelength shift as a function of temperature with linear fitting for the three sensors. The magnified spectra show the wavelength shift of the main reflection band with increasing temperature, which was used for temperature calibration.

Figure 3 shows the temperature response of three cleaved-CFBG sensors immersed in water and heated from 25 °C to 80 °C in 5 °C steps. Increasing temperature causes a shift of the entire spectral profile along the wavelength axis, as seen in the magnified view. As can be seen in the magnified spectrum of Sensor 1, the Bragg peak shifts towards longer wavelengths with increasing temperature, which enables for temperature sensitivity analysis via wavelength tracking. The reflected power remains, however, almost constant, with traces aligned at the same vertical level. The evenly spaced peaks indicate a linear temperature response. The spectra of Sensor 2 and Sensor 3 show a similar shift of the reflection band towards higher wavelengths with increasing temperature. Their reflection bands are, however, broader and do not feature a clear peak, unlike Sensor 1. This prevents the use of straightforward peak-tracking techniques. Instead, a practical alternative is to define a horizontal reference line 3–5 dB below the main reflection band and track the intersection points with each spectrum to determine wavelength shifts. Like Sensor 1, these spectra also show stable vertical alignment, indicating consistent reflected power across the tested temperature range.

A linear relationship between temperature and wavelength shift was confirmed through calibration experiments, shown in Figure 3. A linear fit was applied to estimate the temperature sensitivity (∆λ/∆T) for all three sensors, using the spectrum at 25 °C as a reference. According to Figure 3, the thermo-refractometers show similar temperature sensitivities: 9.357 pm/°C for Sensor 1, 10.64 pm/°C for Sensor 2 and 10.05 pm/°C for Sensor 3. This indicates that the residual grating length can affect the thermal response. Overall, all CFBGs demonstrate sensitivity close to the typical value of around 10 pm/°C reported in different studies [3,9,27].

### 3.3. Refractive Index Sensitivity Analysis

In this section, the results of the RI calibration of the sensors are reported. The RI calibration focused on analyzing sidelobe intensity changes when sensors were exposed to sucrose solutions ranging from 10% to 15% sucrose, such that the RI varies from 1.3285 to 1.3377. The RI is referenced at a wavelength around 1550 nm [36]. The RIs of sucrose solutions have been reported over a wide concentration range from 0% to 65%, showing an increase from approximately 1.31 to 1.44 [37]. Based on these reported data, the RI variation over limited concentration intervals can be considered approximately linear, including the 10–15% range used in this work. Therefore, the RI values were calculated by linearly adding the concentration-dependent RI increment of 1.85 × 10^−3^ per 1% sucrose concentration, as obtained in our previous study. This gives an RI range of 1.3285–1.33775 for the investigated sucrose concentrations, measured in increments of 0.5% around 1550 nm [36].

In addition to this primary RI range, the sensors were evaluated over a broader sucrose concentration range from 0% to 35% with 5% increments; for this extended RI calibration, the refractive indices of sucrose solutions were determined using a third-order polynomial relationship for aqueous sucrose solutions at n = 1550 nm reported in [38]:

(6) $$n_{1550}=Aw^{3}+Bw^{2}+Cw+D,$$

where *w* is the sucrose mass fraction, and *A* = 0.0330, *B* = 0.0456, *C* = 0.1416, and *D* = 1.3166 are the corresponding polynomial coefficients.

The resulting RI values are summarized in Table 3. These values were used to additionally evaluate the sensors’ responses over a broader concentration range, while the detailed RI-sensitivity analysis was performed within the 10–15% sucrose range, consistent with previous studies [36,37].

Over the broader concentration interval, both the concentration–RI relationship and the sensor response become increasingly nonlinear. Therefore, the extended 0–35% dataset was not treated as a single linear calibration range. Instead, it was used to verify that the RI-dependent sidelobe response was preserved over a substantially broader experimentally investigated interval.

The RI values used in these calibration experiments were calculated from the published concentration-dependent relationships rather than measured directly using an independent refractometer. Since RI depends on wavelength, RI values reported at substantially different optical wavelengths cannot be directly transferred to the present measurements around 1550 nm without dispersion correction. Therefore, wavelength-specific relationships reported at 1550 nm were used where available. Nevertheless, the use of calculated rather than directly measured RI values represents an additional source of uncertainty associated with solution preparation, temperature, and the reference relationship.

In addition, it should be noted that the RI values in the focused 10–15% calibration and in the extended 0–35% dataset were obtained using two different literature-based relationships. The focused calibration retains the relationship used in our previous study to preserve consistency with the earlier quantitative analysis, whereas the extended dataset uses the wavelength-specific polynomial at 1550 nm reported in [38]. Consequently, small differences occur between the RI values assigned to overlapping sucrose concentrations.

**Figure 4:**
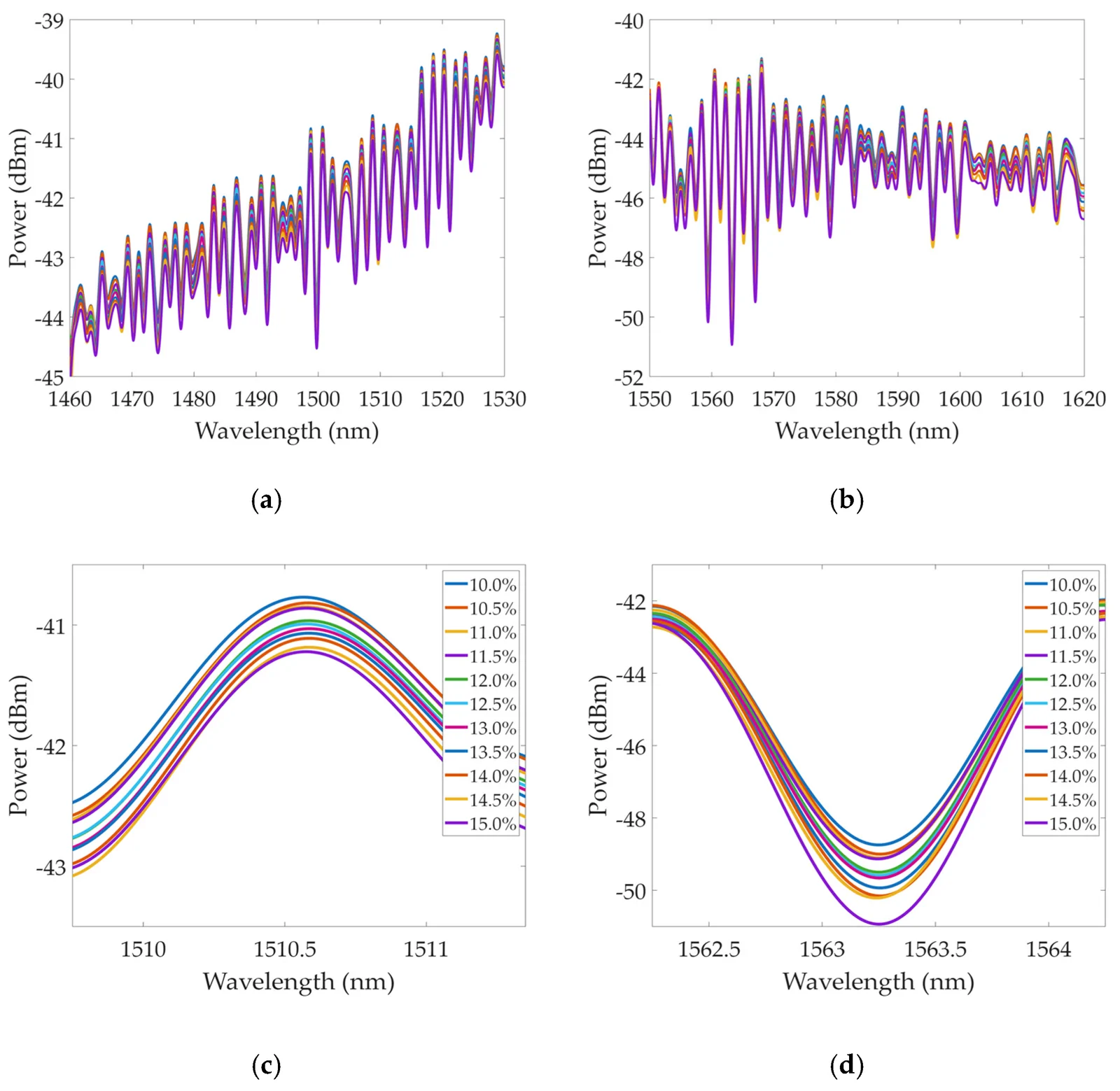
Sidelobe spectral response of Sensor 1 with a residual grating length of 3 mm during RI calibration in sucrose solutions: (**a**) left-sidelobe region; (**b**) right-sidelobe region; (**c**) representative example of a sidelobe peak showing intensity attenuation with increasing RI in the focused 10% to 15% range; (**d**) representative example of a sidelobe valley showing intensity attenuation with increasing RI in the focused 10% to 15% range.

Figure 4 illustrates the spectral output of the sidelobes for Sensor 1 in the 1470–1530 nm left-sidelobe and 1560–1620 nm right-sidelobe ranges. It can be seen that the response does not form a perfect cavity pattern, since the residual CFBG produces a more irregular spectral pattern. However, the RI-induced attenuation remains consistent across the selected sidelobe features, which allows the intensity-based response to be used for calibration. This confirms that the sidelobe intensity can be used as an RI-sensitive metric. When the sensors are exposed to sucrose solutions with increasing concentrations, the RI calibration findings for Sensors 2 and 3 show a similar trend, with continuous attenuation of the sidelobe power.

The RI-dependent intensity response originates from the interaction between the distributed reflection of the residual CFBG and the weak reflection at the cleaved silica–solution interface. A change in the external RI modifies the reflection coefficient at the cleaved tip and therefore changes the relative amplitude of the optical components contributing to the post-cleavage sidelobe pattern. Since the CFBG is chirped, different wavelengths are predominantly reflected from different longitudinal positions of the remaining grating, and truncation consequently produces a wavelength-dependent and asymmetric spectral response. This explains why the magnitude of the RI sensitivity varies substantially between individual sidelobe features and between the lower- and higher-wavelength sides of the spectrum.

**Figure 5:**
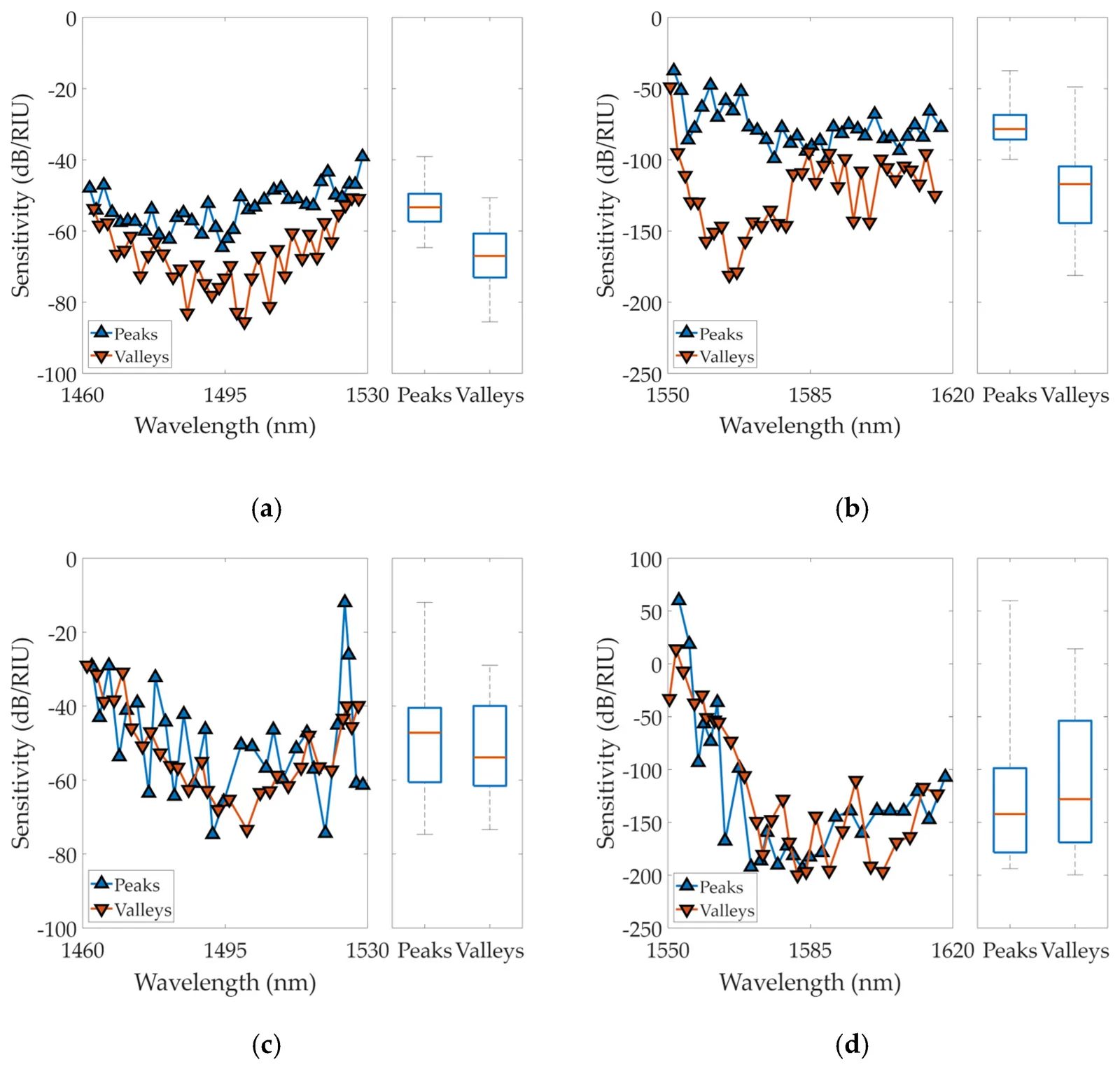

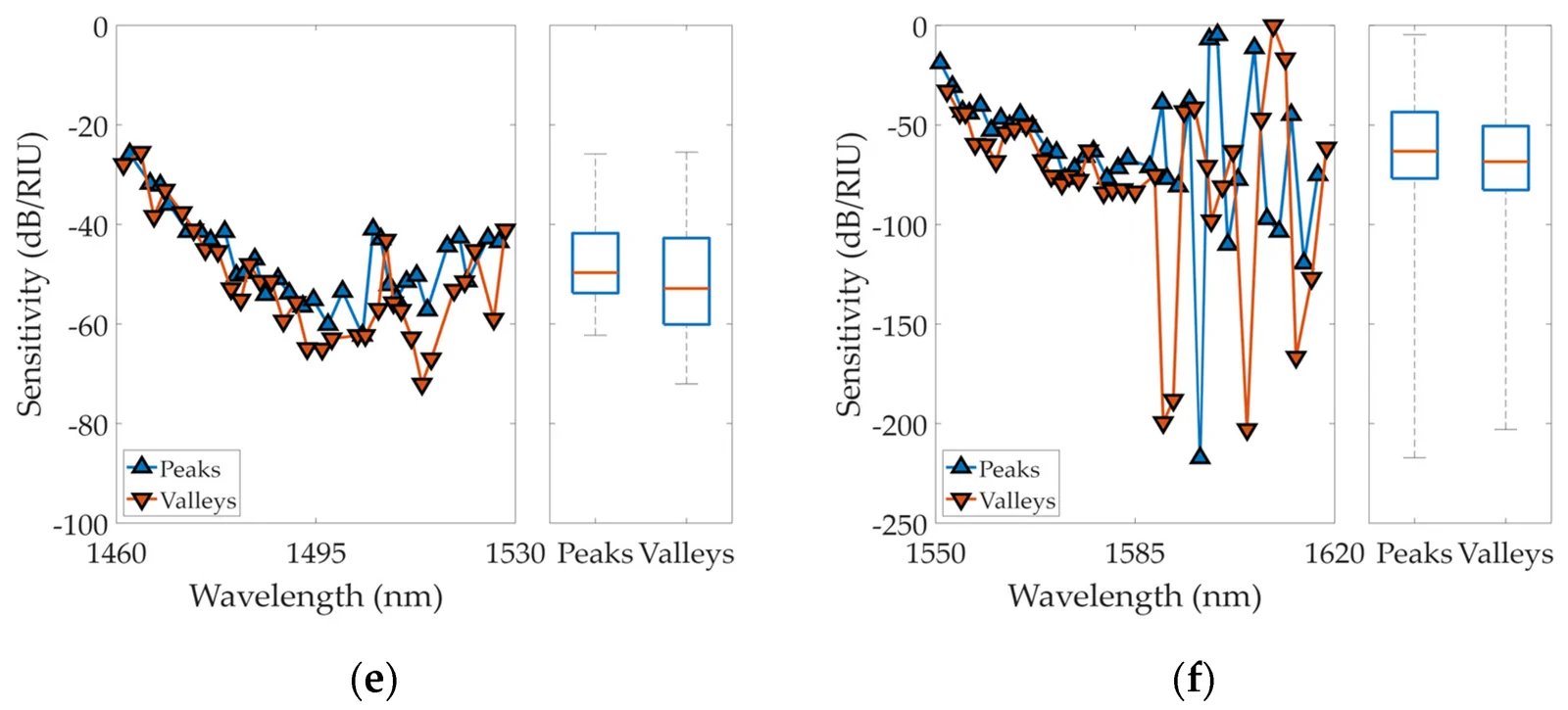
Distribution of RI intensity sensitivity extracted from all detected sidelobe peaks and valleys of the three cleaved-CFBG sensors: (**a**) and (**b**) show the left and right sidelobe regions for Sensor 1 with a residual grating length of 3 mm, respectively; (**c**) and (**d**) show the left and right sidelobe regions for Sensor 2 with a residual grating length of 4 mm, respectively; (**e**) and (**f**) show the left and right sidelobe regions for Sensor 3 with a residual grating length of 5 mm, respectively.

Sensitivity of thermo-refractometers to RI variations was evaluated at each spectral peak and valley on the left and right sidelobes individually. For each fabricated sensor, the detected peaks and valleys were first evaluated individually by linear regression of reflected intensity against RI. Candidate interrogation features were compared according to the magnitude of the fitted RI sensitivity and the corresponding regression quality. Because the post-cleavage sidelobe pattern depends on the individual sensor geometry, a single spectral feature or fixed wavelength cannot be defined a priori as optimal for all fabricated sensors. For every extremum, a linear regression was performed between reflected power—I and the RI—n. The slope of each regression line represents the local RI sensitivity as the ratio of (∆I/∆n), and these values are plotted for Sensor 1, Sensor 2, and Sensor 3 in Figure 5. All three sensors show a non-uniform distribution of sensitivity along the wavelength axis. The sensitivity values vary strongly between adjacent peaks and valleys due to the complex interference pattern generated within the semi-distributed cavity. In the present sensors, several features in the right-hand, higher-wavelength sidelobe region exhibited larger RI sensitivities than those on the left-hand side. This asymmetry is attributed to the wavelength-dependent reflection distribution of the truncated chirped grating and should be regarded as a characteristic of the fabricated sensors rather than as a universal property of cleaved CFBGs.

Sensor 1 shows sensitivities ranging from about −40 dB/RIU to −85 dB/RIU on the left sidelobe and down to −180 dB/RIU on the right sidelobe. Peaks consistently provide lower sensitivities in terms of magnitude than valleys. Meanwhile, Sensor 2 exhibits moderate sensitivities, typically between −10 dB/RIU and −90 dB/RIU in the left region and −10 dB/RIU and −200 dB/RIU on the right region. The greater spread of values compared with Sensor 1 indicates a higher degree of spectral asymmetry and mode field variation along the cleaved grating. The results for Sensor 3 demonstrate the most stable and highest sensitivities with values mostly in the −25 dB/RIU to −75 dB/RIU range on the left sidelobes and reaching about −220 dB/RIU on the right sidelobes.

All sensors show several sensitivity maxima and minima at alternating peaks and valleys. Among them, Sensor 3 shows the highest overall sensitivity in both regions. This can be due to optimized residual grating length and cavity structure, which enhance the field overlaps with the surrounding medium. The RI sensitivity is strongly dependent on the particular spectral feature and sensor geometry. Both peaks and valleys can exhibit pronounced RI-dependent intensity responses. In the present measurements, valley features provided higher sensitivities for Sensors 1 and 2, whereas Sensor 3 exhibited a selected peak sensitivity of −217.2 dB/RIU compared with −202.9 dB/RIU for the corresponding valley. Therefore, neither peaks nor valleys can be considered universally more sensitive. For the subsequent cross-sensitivity analysis, valley features were selected consistently across all three sensors to facilitate direct comparison, while recognizing that peak-based interrogation may provide the highest sensitivity for a particular fabricated sensor. From a design perspective, these results confirm that the RI sensitivity is spectrally localized, and careful selection of wavelength windows allows precise and repeatable interrogation of the RI. The consistent linear regression fits (R^2^ > 0.98 for all extracted slopes) further validate the linear and stable response of the sensors to RI changes.

Across the three investigated residual grating lengths, the sensors exhibited the same general sensing principle but different detailed spectral responses and sensitivity distributions. These differences indicate that the post-cleavage response depends not only on the nominal residual grating length but also on device-specific fabrication variations. Therefore, the present three-sensor comparison should not be interpreted as establishing a universal dependence of sensitivity on residual grating length. A systematic study involving multiple independently fabricated sensors at each residual length would be required to quantify such a relationship.

**Figure 6:**
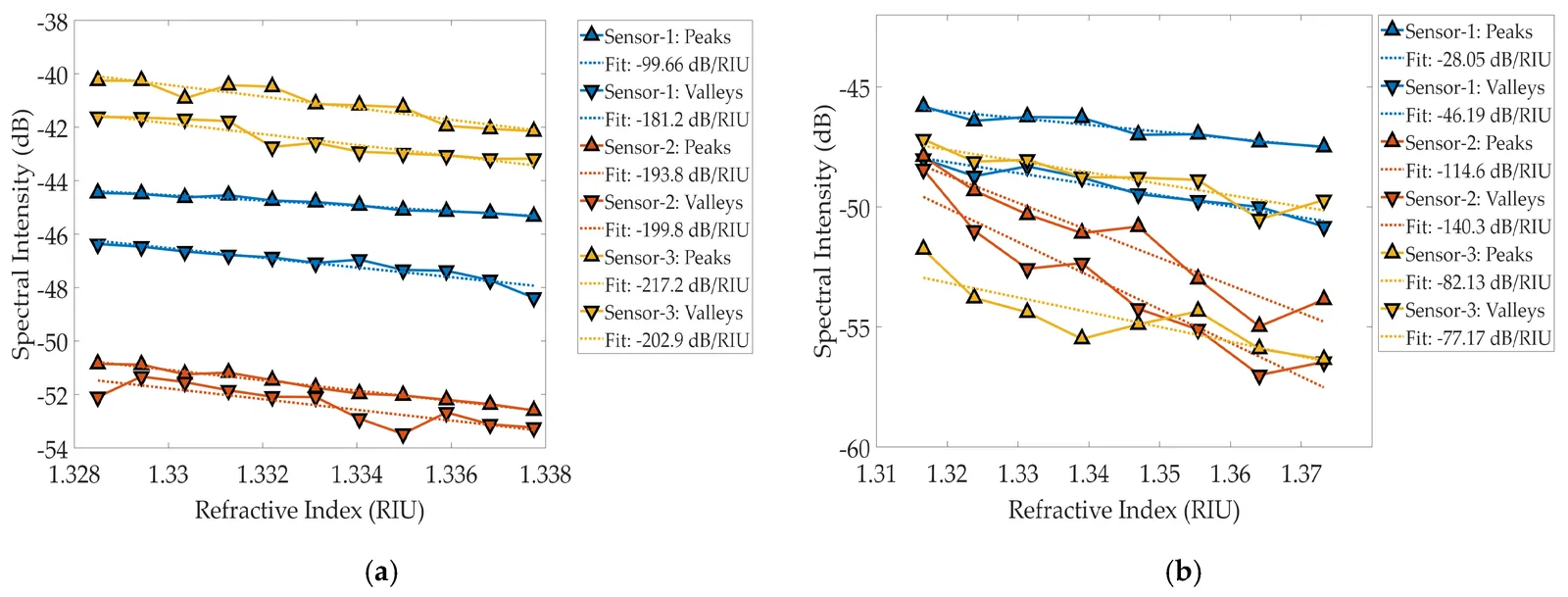
Intensity responses of selected sidelobe peaks and valleys as a function of RI for three sensors: (**a**) primary sensitivity analysis within the 10–15% range of sucrose solutions, and (**b**) extended RI responses corresponding to 0–35% sucrose solutions.

For completeness, Figure 6 presents the intensity responses of the selected sidelobe peaks and valleys as a function of RI for all three sensors over the primary calibration interval and the extended sucrose concentration range. Within the focused 10–15% calibration range shown in Figure 6a, the peak and valley intensities exhibit an approximately linear decrease with RI (1.3285–1.33775 RIU). Linear fits give sensitivities of −99.66/−181.2 dB/RIU for Sensor 1, −193.8/−199.8 dB/RIU for Sensor 2, and −217.2/−202.9 dB/RIU for Sensor 3 for the selected peak and valley features, respectively. These spectral features were therefore used to estimate RI sensitivity during sucrose-solution calibration.

Figure 6b shows the corresponding responses obtained over the extended 0–35% sucrose concentration range. Across this broader RI interval, the selected peak and valley intensities generally decrease with increasing RI, confirming that the RI-dependent sidelobe response is maintained beyond the primary calibration range. However, the responses exhibit greater deviations from linearity than those observed within the narrower 10–15% interval, with fitted sensitivities of −28.05/−46.19 dB/RIU for Sensor 1, −114.6/−140.3 dB/RIU for Sensor 2, and −82.13/−77.17 dB/RIU for Sensor 3.

The larger deviations for the wider concentration range may be ascribed to the nonlinear response inherent to the wider RI range and the larger experimental variation due to the larger concentration increments and the sequential addition of the stock solution. As the sucrose concentration was progressively increased, the total solution volume also changed, while each measurement required mixing and stabilization before spectral acquisition. These effects may contribute to greater variability over the extended range compared with the focused 10–15% calibration interval. For this reason, the extended dataset was used primarily to confirm preservation of the RI-dependent response over a broader range rather than to redefine the quantitative sensitivity obtained from the narrower calibration interval.

### 3.4. Cross-Sensitivity Analysis

Cross-sensitivity analysis was also conducted to check whether the variations in temperature and RI significantly affect the intensity and wavelength shifts, respectively, which should be negligible compared to primary sensitivities.

**Figure 7:**
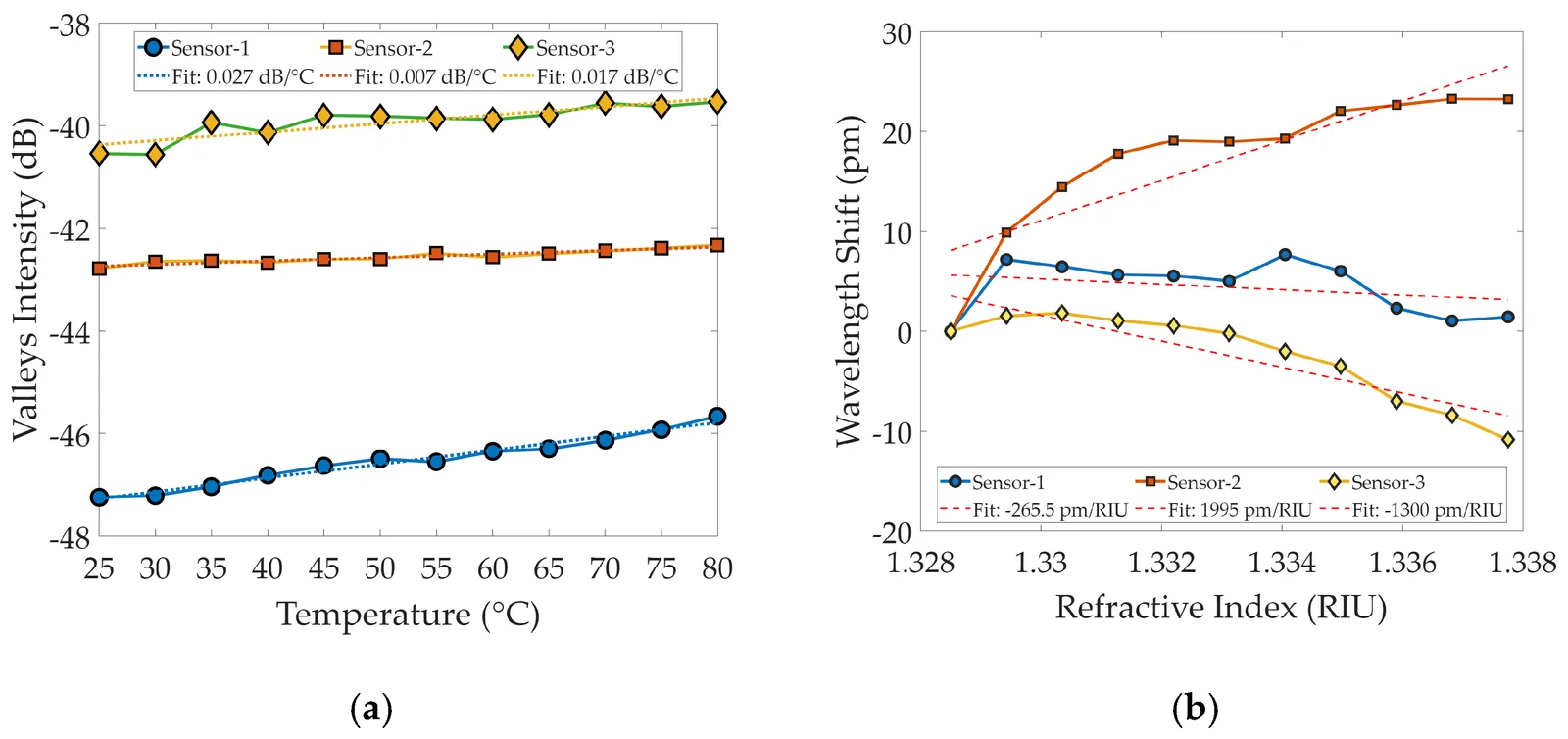
Cross-sensitivity characterization of the three cleaved-CFBG sensors: (**a**) temperature-induced intensity variation of the RI-sensitive sidelobe valley features; (**b**) RI-induced wavelength shift of the temperature-sensitive main reflection band.

Figure 7a shows the dependence of valley intensity on temperature in the range of 25–80 °C. All three sensors exhibit minimal and nearly linear intensity variation with increasing temperature. The linear regression fits yielded slopes of 0.027 dB/°C for Sensor 1, 0.007 dB/°C for Sensor 2, and 0.017 dB/°C for Sensor 3. These small slopes confirm that temperature fluctuations cause negligible changes in measured intensity. This means that the RI-sensitive sidelobes remain stable and largely unaffected by temperature variations.

Figure 7b demonstrates the wavelength shift of the main band as a function of RI. The variation in RI across the calibration range (1.328–1.338 RIU) corresponds to a change of only about 0.01 RIU, resulting in very small absolute wavelength shifts, typically on the order of a few picometers. Linear regression analysis produced slopes of −265.5 pm/RIU for Sensor 1, 1995 pm/RIU for Sensor 2, and −1300 pm/RIU for Sensor 3. Although Sensor 2 shows a higher (Δλ/Δn) slope, this corresponds to only minor wavelength changes over the narrow RI interval and is therefore not practically significant. Moreover, the relatively low R^2^ values indicate weak or nonlinear correlations between the values.

Mutual dependence between RI and temperature responses can be quantitatively evaluated using a cross-sensitivity matrix approach, where the temperature (∆λ/∆T) and RI (∆I/∆n) sensitivities are positioned on the main diagonal, while the non-diagonal elements represent the cross-sensitivities (∆I/∆T and ∆λ/∆n). The obtained results are used to construct the cross-sensitivity matrices for the sensors according to Equation (1), as shown in Equations (7)–(9). Previously found RI sensitivities show that valleys tend to be more sensitive to RI variations compared to peaks. However, both peak and valley features can provide RI-sensitive responses. In the present measurements, valleys provided the higher selected sensitivity for Sensors 1 and 2, whereas Sensor 3 exhibited a higher peak sensitivity (−217.2 dB/RIU) than valley sensitivity (−202.9 dB/RIU). For consistency of the matrix comparison across the three sensors, valley-based sensitivities were nevertheless used throughout the cross-sensitivity analysis. This choice does not imply that valley interrogation is intrinsically superior to peak interrogation.

Based on the obtained RI, temperature and cross-sensitivity coefficients shown in Figure 6 and Figure 7, the sensitivity matrices for the three sensors can be written as follows.

For Sensor 1 (3 mm):

(7) $$\left[\begin{array}{c} ∆I\\ ∆\lambda \end{array}\right]=\left[\begin{array}{cc} -181.2\;dB/RIU) & 0.027\;dB/{}^{\circ}C\\ -265.5\;pm/RIU & 9.357\;pm/{}^{\circ}C \end{array}\right]\left[\begin{array}{c} ∆n\\ ∆T \end{array}\right],$$

For Sensor 2 (4 mm):

(8) $$\left[\begin{array}{c} ∆I\\ ∆\lambda \end{array}\right]=\left[\begin{array}{cc} -199.8\;dB/RIU) & 0.007\;dB/{}^{\circ}C\\ 1995\;pm/RIU & 10.64\;pm/{}^{\circ}C \end{array}\right]\left[\begin{array}{c} ∆n\\ ∆T \end{array}\right],$$

For Sensor 3 (5 mm):

(9) $$\left[\begin{array}{c} ∆I\\ ∆\lambda \end{array}\right]=\left[\begin{array}{cc} -202.9\;dB/RIU) & 0.017\;dB/{}^{\circ}C\\ -1300\;pm/RIU & 10.05\;pm/{}^{\circ}C \end{array}\right]\left[\begin{array}{c} ∆n\\ ∆T \end{array}\right],$$

These matrices have RI and temperature sensitivities on the main diagonal and cross-sensitivity terms in the off-diagonal elements. The determinant of each matrix is compared to the product of its main diagonal elements to assess these cross-sensitivities. For Sensor 1 (3 mm), the determinant is −1688.32, whereas the product of the main diagonal is −1695.49, giving a difference of only about 0.42%. For Sensor 2 (4 mm), these values are −2139.84 and −2125.87, respectively, corresponding to a difference of about 0.66%. For Sensor 3 (5 mm), the determinant is −2017.05 compared with −2039.15 for the main diagonal product, giving a difference of about 1.08%. Since these differences remain close to 1% or lower, the contribution of the cross-sensitivity terms is small compared with the main sensing terms. This indicates that RI and temperature responses are approximately decoupled for all three sensors. Accordingly, the measured cross-sensitivity coefficients suggest that measurements of RI at different temperatures, or temperature at different RI values, would introduce only minor deviations from the independently obtained calibration responses within the investigated ranges. Nevertheless, experimentally determined cross-sensitivity terms are retained in the matrix inversion to account for the residual coupling between the two measurands. The corresponding inverse sensitivity matrices are given in Equations (10)–(12).

For Sensor 1 (3 mm):

(10) $$\left[\begin{array}{c} ∆n\\ ∆T \end{array}\right]=\left[\begin{array}{cc} -5.542\times 10^{-3}\;RIU/dB) & 1.599\times 10^{-5}\;RIU/pm\\ -0.1573\;{}^{\circ}C/dB & 0.1073\;{}^{\circ}C/pm \end{array}\right]\left[\begin{array}{c} ∆I\\ ∆\lambda \end{array}\right],$$

For Sensor 2 (4 mm):

(11) $$\left[\begin{array}{c} ∆n\\ ∆T \end{array}\right]=\left[\begin{array}{cc} -4.972\times 10^{-3}\;RIU/dB) & 3.271\times 10^{-6\;}RIU/pm\;\\ 0.9323\;{}^{\circ}C/dB & 0.0934\;{}^{\circ}C/pm \end{array}\right]\left[\begin{array}{c} ∆I\\ ∆\lambda \end{array}\right],$$

For sensor 3 (5 mm):

(12) $$\left[\begin{array}{c} ∆n\\ ∆T \end{array}\right]=\left[\begin{array}{cc} -4.983\times 10^{-3}\;RIU/dB) & 8.428\times 10^{-6}\;RIU/pm\;\\ -0.6445\;{}^{\circ}C/dB & 0.1006\;{}^{\circ}C/pm \end{array}\right]\left[\begin{array}{c} ∆I\\ ∆\lambda \end{array}\right],$$

For all three sensors, the RI reconstruction is dominated by the intensity term, with coefficients of −5.542×10^−3^, −4.972×10^−3^, and −4.983×10^−3^ RIU/dB for Sensors 1–3, respectively. Similarly, the temperature reconstruction is mainly determined by the wavelength response, with coefficients of 0.1073, 0.0934, and 0.1006 °C/pm. The remaining terms provide a correction for the weak coupling between RI and temperature responses. Thus, the inverse matrices provide the mathematical relation required for simultaneous reconstruction of both parameters while accounting for the measured cross-sensitivity.

### 3.5. Simultaneous Parameter Reconstruction

To experimentally evaluate the inverse sensitivity matrix under simultaneous changes of both measurands, the three sensors were tested under six combined conditions: 25 °C/10%, 30 °C/15%, 35 °C/20%, 40 °C/25%, 45 °C/30%, and 50 °C/35% sucrose concentration. The first condition 25 °C/10% was taken as the reference state. For each subsequent condition j, the wavelength and intensity changes were calculated relative to this reference as ${∆\lambda }_{j}=\lambda_{j}-\lambda_{1}$ and ${∆I}_{j}=I_{j}-I_{1}$.

**Figure 8:**
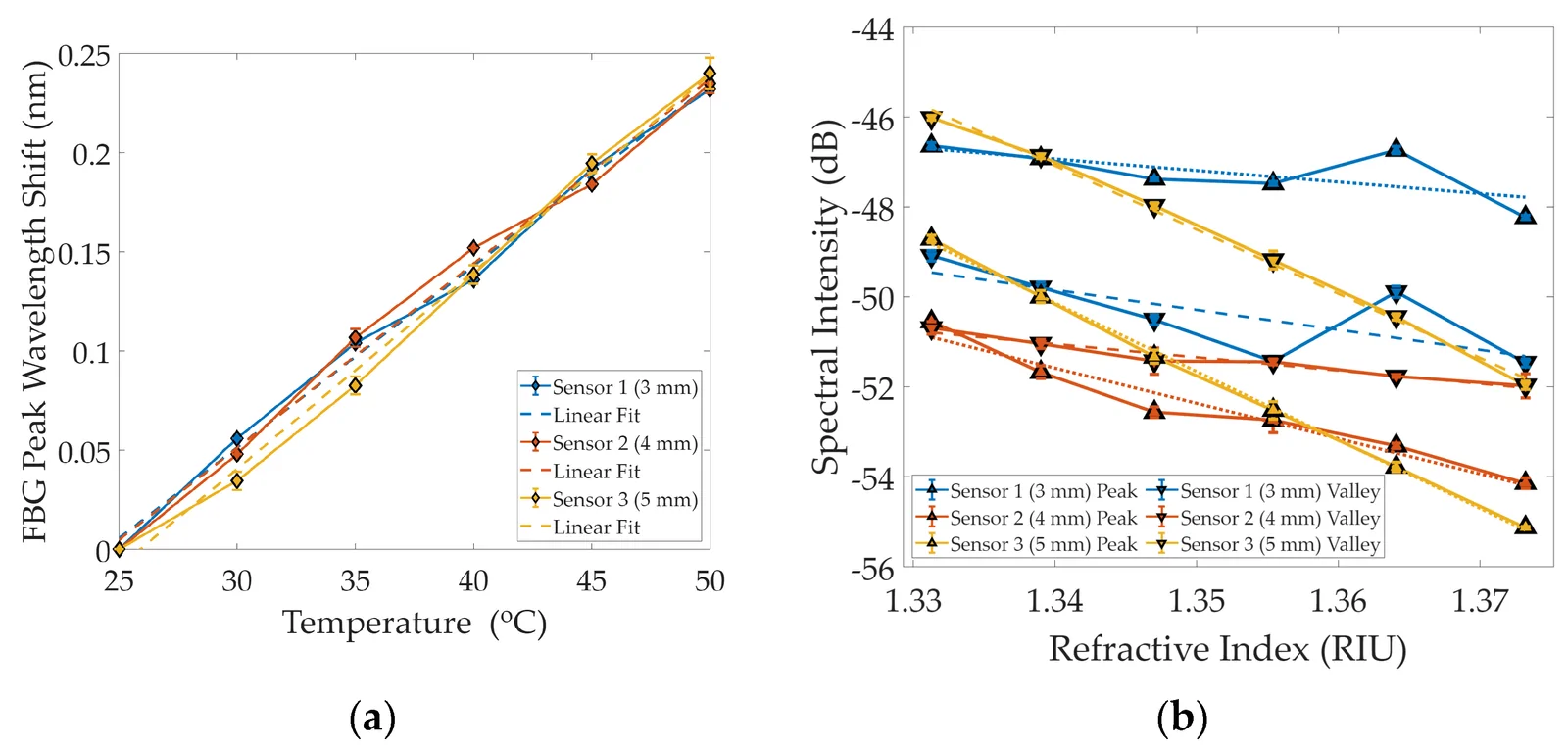
Measured response of the three sensors under combined temperature and sucrose concentration conditions of 25 °C/10%, 30 °C/15%, 35 °C/20%, 40 °C/25%, 45 °C/30%, and 50 °C/35%: (**a**) main-band wavelength shift versus temperature; (**b**) selected sidelobe peak and valley spectral intensities versus RI.

Figure 8 shows the measured wavelength shift and intensity of the responses under these combined conditions. The wavelength shift increased with rising temperature and sucrose concentration for all three sensors, although the magnitude of the shift varied between sensors. The valley intensity response generally decreased across the investigated conditions with greater variation between the sensors. These experimentally measured changes were used as inputs to the corresponding inverse sensitivity matrices for parameter reconstruction.

For each sensor, the measured ∆λ and ∆I values were substituted into the corresponding inverse sensitivity matrix yielding the reconstructed changes in both temperature and RI. The reconstructed temperature change was added to the reference temperature of 25 °C and compared with the experimentally imposed temperature. Although the inverse matrix also provides the corresponding RI change, quantitative validation of the reconstructed RI requires reference RI values for each sucrose solution at its actual experimental temperature and at the sensing wavelength. Since such reference values were not independently available for the present measurements, only temperature reconstruction was used for the quantitative error analysis. Published RI data are frequently reported at other wavelengths and/or temperatures, and their direct application to measurements around 1550 nm would require additional wavelength- and temperature-dependent compensation. For this reason, no literature-derived RI values were introduced solely for the purpose of validating the reconstruction. The reconstructed temperatures are summarized in Table 4.

Reconstruction accuracy was further assessed using the Root Mean Square Error (RMSE). The reference condition was excluded from the analysis because its zero-reconstruction error is imposed by the definition and would artificially reduce the overall error estimate. The temperature RMSE values were 0.87, 2.69, and 1.86 °C for Sensors 1, 2 and 3, respectively. Sensor 1 showed the closest agreement with the reference temperatures, with a maximum absolute deviation of 1.39 °C. Larger deviations were observed for Sensors 2 and 3 toward the upper end of the tested range, where Sensor 2 increasingly underestimated and Sensor 3 overestimated the temperature.

These deviations may partly be related to differences in the thermal conditions between the initial temperature calibration and the simultaneous measurements. The temperature calibration was performed in a relatively large volume of water, where the temperature changed more gradually and could be allowed to stabilize before each spectral acquisition. In contrast, the simultaneous measurements were carried out using a much smaller volume of sucrose solution which underwent more rapid temperature changes and made it more difficult to maintain thermal equilibrium during the measurements. Consequently, the temperature indicated by the thermocouple may not have exactly represented the temperature experienced by the sensing region at the time of spectral acquisition. Transient temperature nonuniformity within the solution and the finite response time of the thermocouple may have further contributed to this mismatch. Since the temperature sensitivity coefficients were obtained under comparatively stable calibration conditions, their application under these more transient thermal conditions may partly account for the observed reconstruction errors. Accordingly, the reconstruction results should be regarded as an experimental validation of the inverse-matrix approach rather than as a complete accuracy characterization of the sensor under fully controlled simultaneous variations of temperature and RI. A complete validation of simultaneous two-parameter reconstruction will therefore require a two-dimensional calibration grid in which several RI values are independently tested at each of several fixed temperatures.

### 3.6. Biomarker Detection

After biofunctionalization with anti-VEGF antibodies, the cleaved-CFBG sensors were evaluated as label-free biosensors for VEGF detection. In contrast to the sucrose-based RI calibration, where the response originates from bulk RI changes, the VEGF assay relies on local RI variations at the antibody-modified fiber tip caused by biomolecular binding. The response was monitored through the intensity changes of the sidelobe peaks and valleys, since these spectral features were previously identified as the RI-sensitive regions of the cleaved-CFBG spectrum.

**Figure 9:**
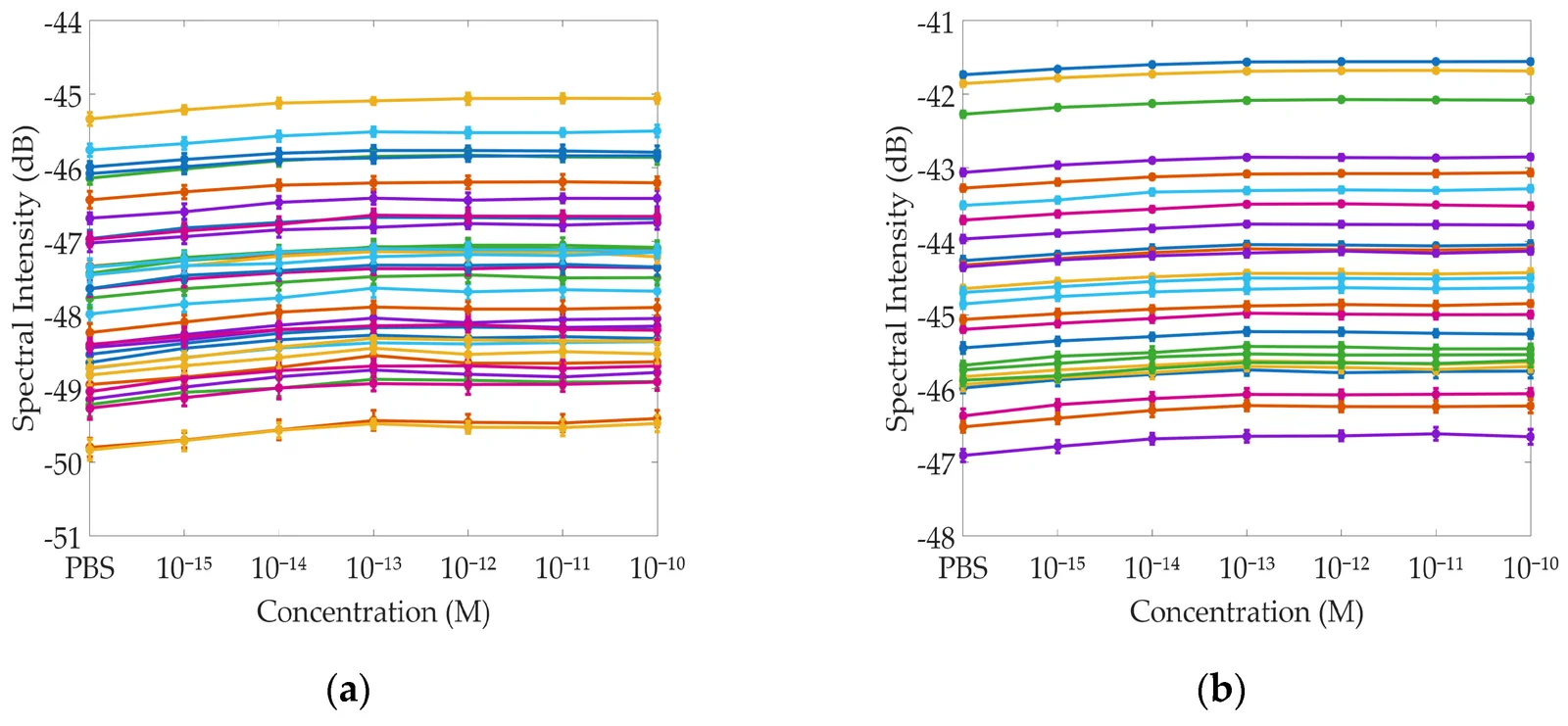

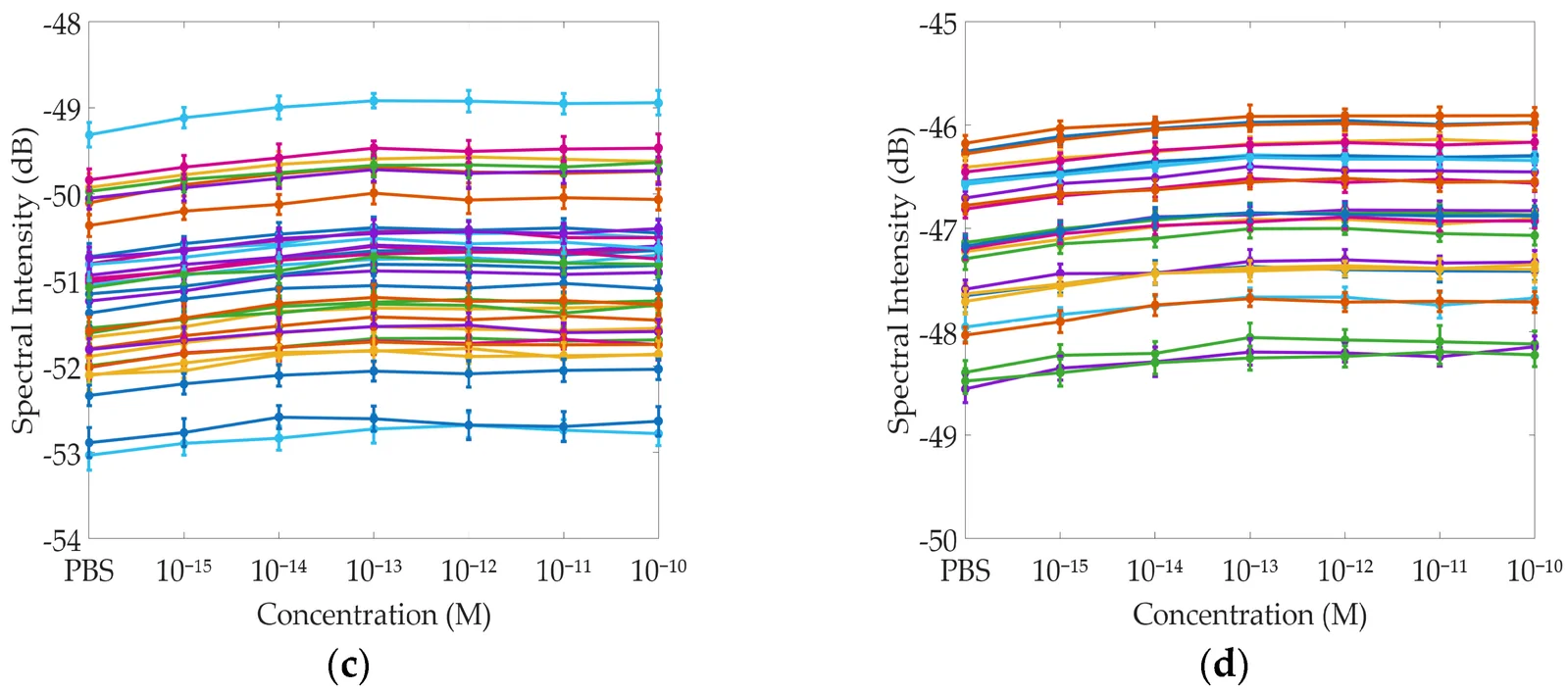
VEGF concentration response of the anti-VEGF-functionalized cleaved-CFBG sensor based on all detected sidelobe peaks and valleys: (**a**) left-sidelobe peaks; (**b**) right-sidelobe peaks; (**c**) left-sidelobe valleys; (**d**) right-sidelobe valleys. The responses are shown from PBS to VEGF concentrations ranging from 10^−15^ M to 10^−10^ M.

Figure 9 summarizes the spectral intensity response of the functionalized sensor as a function of VEGF concentration from 10^−15^ M to 10^−10^ M. Most of the analyzed peaks and valleys show a measurable intensity increase at low VEGF concentrations, especially between the blank and the 10^−15^–10^−13^ M range, followed by a tendency toward signal stabilization at higher concentrations. At higher concentrations, the response becomes progressively less pronounced, indicating a saturation-like behavior. However, the present measurements do not allow the origin of this response flattening to be uniquely identified.

**Figure 10:**
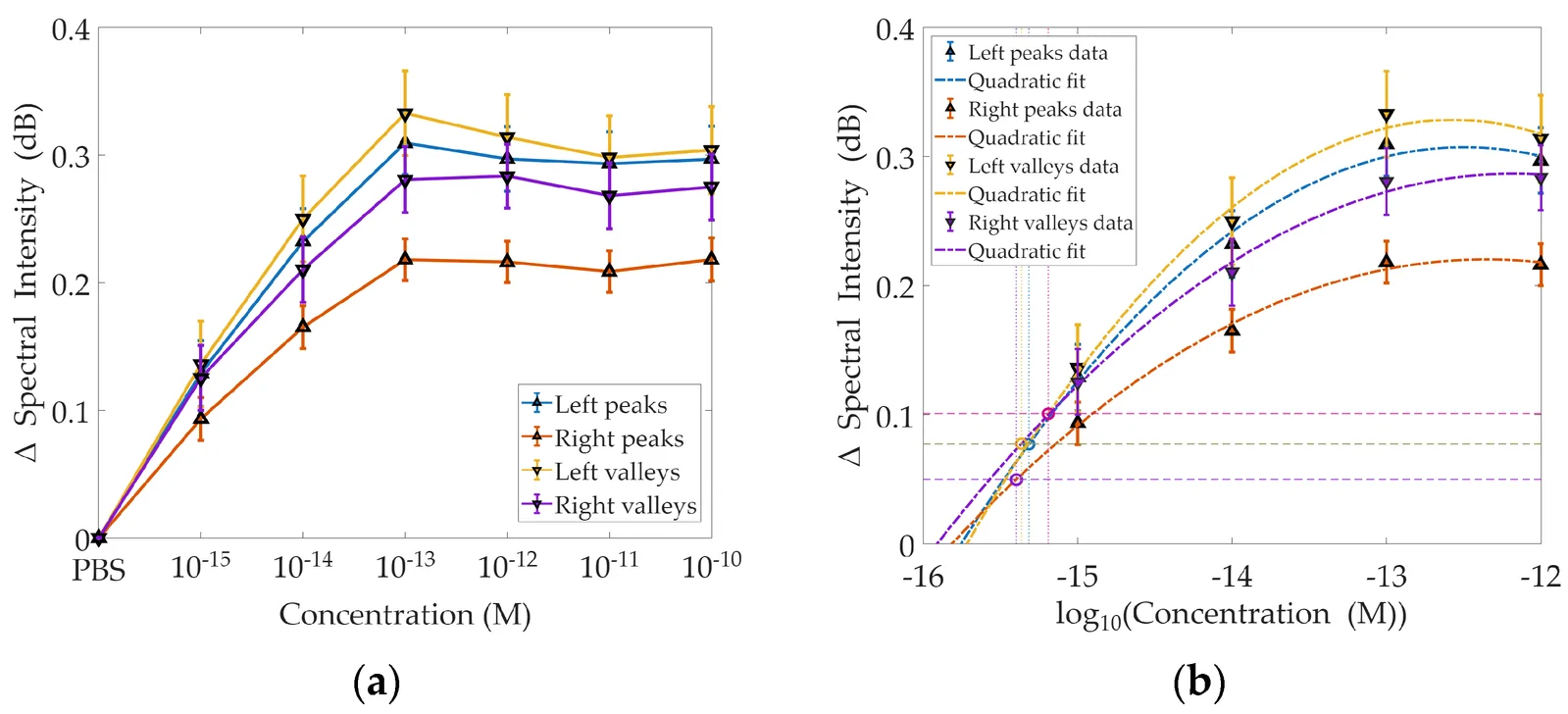
VEGF detection responses and LoD estimation: (**a**) concentration-dependent mean intensity changes of the left and right sidelobe peaks and valleys relative to the PBS blank; (**b**) low-concentration and LoD estimation using second-order polynomial fit.

Figure 10 summarizes the concentration-dependent response of the functionalized cleaved-CFBG sensor by averaging all detected spectral extrema shown in Figure 9. For each VEGF concentration, the responses of all left peaks, right peaks, left valleys, and right valleys were averaged separately after baseline correction with respect to the PBS condition. The largest response variation occurs within the low-concentration region, whereas the response becomes progressively less pronounced at higher VEGF concentration.

For LoD estimation, the first four VEGF concentrations from 10^−15^ M to 10^−12^ M were selected to describe the low-concentration calibration region. Following previously reported approaches for optical fiber biosensors, these data were fitted using a second-order polynomial as a function of ${log}_{10}(C)$ [34,35]. The LoD threshold was defined as $y_{blank}+3\sigma_{max}$, where the PBS-referenced blank response was zero and $\sigma_{max}$ represents the maximum standard deviation obtained from the repeated measurements within the fitted concentration range. The LoD was then determined from the intersection between this threshold and the fitted calibration curve.

The second-order polynomial fits provided R^2^ values of about 0.99 for all spectral features. The corresponding σ_max,_ 3σ_max_ and calculated LoD values are summarized in Table 5. Among the four analyzed spectral-response groups, the right peaks provided the lowest estimated LoD of 3.998 × 10^−16^ M, whereas the left valleys resulted in the highest value of 6.455 × 10^−16^ M. The comparable LoD estimates obtained from the different sidelobe regions indicate that the low-concentration response is consistently resolved across both peak- and valley-based interrogation.

The proposed CFBG platform shows a different detection mechanism compared with the previously reported VEGF biosensor based on the SDI approach [32]. In this configuration, VEGF binding is mainly resolved through the intensity changes of the sidelobes, while the Bragg band remains available for temperature monitoring within the same fiber structure. Therefore, these results support the feasibility of the proposed cleaved-CFBG sensor as a dual parameter platform for preliminary VEGF biosensing. However, additional independent sensor replicates, reference measurements, non-target protein controls and long-term stability tests are required for a more complete assessment of analytical repeatability, selectivity, and reproducibility.

### 3.7. Future Work

Although the proposed cleaved-CFBG thermo-refractometric sensor showed promising dual-parameter sensing performance, several aspects require further investigation. The present temperature calibration was performed under controlled laboratory conditions using gradual heating, whereas practical biomedical applications may involve rapid and spatially non-uniform thermal gradients. Future experiments should therefore evaluate the sensor under localized heating, transient temperature changes, and tissue-mimicking environments.

The simultaneous-parameter experiment provided an initial validation of the inverse sensitivity matrix under combined temperature and sucrose concentration changes. In addition, the determinant analysis showed that the contribution of the off-diagonal cross-sensitivity terms was small compared with the primary RI and temperature sensitivities. However, temperature and concentration were varied together along a single sequence rather than independently over a complete two-dimensional temperature–RI calibration grid. Moreover, quantitative reconstruction of RI could not be independently validated because reference RI values corresponding to each sucrose concentration at the actual experimental temperature and sensing wavelength were not available. Future work should therefore perform measurements at several RI values for each of several fixed temperatures, allowing direct validation of both reconstructed parameters and a more rigorous assessment of the stability of the sensitivity matrix.

The RI response was additionally examined over an extended 0–35% sucrose concentration range, whereas quantitative sensitivity analysis remained focused on the 10–15% interval. Although the broader measurements confirmed preservation of the RI-dependent sidelobe response, greater deviations from linearity were observed over the extended range. Additional replicated measurements with improved control of solution composition, temperature, stabilization time, and measurement noise are required to establish the practical linear range, repeatability, and RI detection limit.

Biofunctionalization with anti-VEGF antibodies demonstrated a measurable response to VEGF from 10^−15^ M to 10^−10^ M, and the analytical LoD was estimated from the low-concentration detection using the $y_{blank}+3\sigma_{max}$ criterion. These biosensing results should nevertheless be considered preliminary. Further studies should include independent sensor replicates, reference and negative controls, interfering proteins, systematic selectivity measurements, reproducibility assessment, and long-term stability testing.

Finally, the repeatability of the cleaving procedure should be systematically evaluated, since small variations in residual grating length can modify the resulting sidelobe structure and, consequently, the available RI-sensitive spectral features. Standardization of the fabrication process, together with integration into catheters, needles, endoscopes, or lab-on-chip platforms, will be required to determine the practical applicability of the proposed architecture for biomedical monitoring and biosensing.

## 4. Conclusions

In conclusion, this work demonstrates the development and characterization of a compact cleaved-CFBG sensor for dual temperature and RI measurement, with an additional demonstration of VEGF biosensing after surface biofunctionalization. The sensing structure was fabricated by cleaving a CFBG within the grating region, leaving residual grating lengths of 3 mm, 4 mm, and 5 mm. The detailed post-cleavage sidelobe structure was found to be device-dependent, indicating that future standardization of the fabrication procedure will be required to improve intersensor reproducibility. This modification transformed the original broad CFBG spectrum into a narrower main reflection band accompanied by pronounced sidelobes, enabling wavelength-based temperature interrogation and intensity-based RI interrogation within the same fiber structure.

Temperature calibration from 25 °C to 80 °C produced approximately linear wavelength responses with sensitivities close to the typical CFBG value of 10 pm/°C. RI calibration in the focused 10–15% sucrose range showed a consistent decrease in sidelobe intensity with increasing RI, with selected spectral features exhibiting sensitivities above 200 dB/RIU. Additional measurements over the 0–35% sucrose concentration range confirmed that the RI-dependent response was maintained over a broader interval, although with greater deviation from linear behavior.

The experimentally determined primary and cross-sensitivity coefficients were incorporated into 2 × 2 sensitivity matrices and their inverses to enable simultaneous parameter reconstruction. Comparison of the matrix determinant with the products of the corresponding diagonal sensitivity terms showed differences of approximately 1% or less for all three sensors, indicating a relatively small contribution from the off-diagonal cross-sensitivity terms. Under combined temperature and sucrose concentration variations, reconstructed temperatures followed the imposed temperature trend, with RMSE values of 0.87–2.69 °C across the three sensors. The remaining reconstruction errors are attributed in part to the more transient thermal conditions of the combined experiment and indicate the need for further validation under independently controlled temperature–RI combinations. Quantitative reconstruction of RI under these combined conditions also remains to be experimentally validated using temperature-dependent reference RI values.

After anti-VEGF functionalization, the cleaved-CFBG platform produced concentration-dependent sidelobe intensity changes over the investigated VEGF range from 10^−15^ M to 10^−10^ M, with the response becoming less pronounced at higher concentrations. The LoD was estimated from the low-concentration detection using the $y_{blank}+3\sigma_{max}$ criterion, resulting in values on the order of 10^−16^ M for the analyzed sidelobe features. These results demonstrate the feasibility of extending the same sensing architecture toward label-free biomarker measurements, although the biosensing results should remain preliminary until further validation is performed using independent sensor replicates, reference and negative controls, interfering proteins, selectivity testing, reproducibility analysis, and long-term stability measurements.

Overall, the proposed cleaved-CFBG architecture combines temperature sensing, RI detection, and preliminary biomolecular detection in a compact single-grating structure without chemical etching, tapering, multicore fibers, or additional sensing elements. The results support its potential as a low-complexity thermo-refractometric sensing platform, while further multi-parameter calibration and biosensing validation are required before practical biomedical implementation.

## Figures and Tables

**Table 1:** Comparison of representative fiber optic sensors for simultaneous temperature and RI sensing.

Reference	Sensor Structure	Fabrication or Modification	Temperature Sensing	RI Sensing	Main Advantages and Limitations
Korganbayev et al. [6]	Partially etched CFBG	Partial HF etching of CFBG	Wavelength shift; 31.5–63 °C; ~10.2 pm/°C	Wavelength shift; ΔRI ≈ 0–0.0463; 0.10–1.49 nm/RIU	Enables thermal-profile sensing; requires controlled chemical etching
Kilic et al. [28]	Etched CFBG-FPI in MCF	CFBG inscription in seven-core MCF followed by BHF etching	Central-core notch wavelength shift; 11.9 pm/°C	Differential wavelength shift; ~1.316–1.323 RIU; 1.43 nm/RIU	Strong temperature compensation; relatively complex multicore/cavity fabrication
Ayupova et al. [3]	Shallow-tapered CFBG	CO_2_-laser tapering of CFBG followed by Au coating	Wavelength shift; 30–80 °C; 9.893 pm/°C	Post-taper intensity change; 1.34974–1.35845 RIU; 382.83 dB/RIU	High RI sensitivity and low cross-talk; tapering and coating required
Flores-Bravo et al. [24]	Supermode interferometer	MCF–SMF fusion splicing followed by flat cleaving	Interference-pattern wavelength shift; 0–60 °C; 22.86 pm/°C	FFT amplitude; ~1.30–1.40 RIU; nonlinear calibration	Single reusable probe; requires specialized MCF and FFT processing
Ying et al. [15]	Cascaded tilted-grating/FBG sensor	Cascading of separately fabricated CTFBG and conventional FBG; inscription method not reported	Wavelength shift; 25–300 °C; 13.7–13.9 pm/°C	Wavelength shift; 1.3399–1.3648 RIU; 1.8487 nm/RIU	Effective T–RI decoupling; requires two separate grating elements
Kuang et al. [25]	Tilted-grating reflective probe assisted by CFBG	Femtosecond-laser inscription of FBG, 45–TFG, 81–TFG, and CFBG	Wavelength shift; 30–100 °C; 11.37 pm/°C	Wavelength shift; 1.333–1.375 RIU; up to 516.83 nm/RIU	High RI sensitivity and reflective operation; multi-grating fabrication
This work	Cleaved-CFBG cavity sensor	Cleaving within the CFBG region	Wavelength shift; 25–80 °C; 9.197–10.64 pm/°C	Sidelobe intensity change; 1.3285–1.33775 RIU; up to ~220 dB/RIU	Compact single-CFBG architecture; no etching, tapering, or additional gratings

**Table 2:** Optical fiber parameters.

Parameter	Specification
Center wavelength	1560 ± 0.5 nm
Bandwidth	40 ± 1 nm
Grating length	40 mm
Reflectivity	>90%
Fiber type	SMF-28C
Fiber recoating	acrylate recoating
FBG profile	chirped

**Table 3:** Calculated RI values of aqueous sucrose solutions at 1550 nm.

Sucrose Concentration, (%)	Mass Fraction, W	RI at 1550 nm
0	0.00	1.3166
5	0.05	1.3238
10	0.10	1.3313
15	0.15	1.3390
20	0.20	1.3470
25	0.25	1.3554
30	0.30	1.3641
35	0.35	1.3732

**Table 4:** Temperature reconstruction under the combined experimental conditions.

Reference T (°C)	Sensor 1 Reconstructed T (°C)	Error (°C)	Sensor 2 Reconstructed T (°C)	Error (°C)	Sensor 3 Reconstructed T (°C)	Error (°C)
25	25.00	0.00	25.00	0.00	25.00	0.00
30	31.12	+1.12	29.17	−0.83	29.07	−0.93
35	36.39	+1.39	34.36	−0.64	34.58	−0.42
40	39.97	−0.03	38.49	−1.51	41.05	+1.05
45	45.73	+0.73	41.18	−3.82	47.50	+2.50
50	50.27	+0.27	45.72	−4.28	52.98	+2.98

**Table 5:** LoD estimation parameters obtained from the low-concentration VEGF detection.

Spectral Feature	σ_max_	3σ_max_	R^2^	Estimated LoD
Left peaks	0.02576	0.07729	0.99007	4.840 × 10^−16^
Right peaks	0.01663	0.04990	0.99378	3.998 × 10^−16^
Left valleys	0.03360	0.10080	0.98951	6.455 × 10^−16^
Right valleys	0.02587	0.07760	0.99161	4.316 × 10^−16^

## Data Availability

Data presented in this work are not publicly available at this time. Raw data can be obtained upon reasonable request from the authors.
